# A vascular biology network model focused on inflammatory processes to investigate atherogenesis and plaque instability

**DOI:** 10.1186/1479-5876-12-185

**Published:** 2014-06-26

**Authors:** Héctor De León, Stéphanie Boué, Walter K Schlage, Natalia Boukharov, Jurjen W Westra, Stephan Gebel, Aaron VanHooser, Marja Talikka, R Brett Fields, Emilija Veljkovic, Michael J Peck, Carole Mathis, Vy Hoang, Carine Poussin, Renee Deehan, Katrin Stolle, Julia Hoeng, Manuel C Peitsch

**Affiliations:** 1 Philip Morris International R&D, Philip Morris Products S.A., Quai Jeanrenaud 5, 2000 Neuchâtel, Switzerland; 2 Philip Morris International R&D, Philip Morris Research Laboratories GmbH, Fuggerstr.3, 51149 Koeln, Germany; 3 Selventa, One Alewife Center, Cambridge, MA 02140, USA

**Keywords:** Vascular systems biology, Plaque destabilization, Vascular biology networks, Computational modeling, Atherosclerosis modeling

## Abstract

**Background:**

Numerous inflammation-related pathways have been shown to play important roles in atherogenesis. Rapid and efficient assessment of the relative influence of each of those pathways is a challenge in the era of “omics” data generation. The aim of the present work was to develop a network model of inflammation-related molecular pathways underlying vascular disease to assess the degree of translatability of preclinical molecular data to the human clinical setting.

**Methods:**

We constructed and evaluated the Vascular Inflammatory Processes Network (V-IPN), a model representing a collection of vascular processes modulated by inflammatory stimuli that lead to the development of atherosclerosis.

**Results:**

Utilizing the V-IPN as a platform for biological discovery, we have identified key vascular processes and mechanisms captured by gene expression profiling data from four independent datasets from human endothelial cells (ECs) and human and murine intact vessels. Primary ECs in culture from multiple donors revealed a richer mapping of mechanisms identified by the V-IPN compared to an immortalized EC line. Furthermore, an evaluation of gene expression datasets from aortas of old ApoE^-/-^ mice (78 weeks) and human coronary arteries with advanced atherosclerotic lesions identified significant commonalities in the two species, as well as several mechanisms specific to human arteries that are consistent with the development of unstable atherosclerotic plaques.

**Conclusions:**

We have generated a new biological network model of atherogenic processes that demonstrates the power of network analysis to advance integrative, systems biology-based knowledge of cross-species translatability, plaque development and potential mechanisms leading to plaque instability.

## Background

Evidence gathered from *in vitro* and *in vivo* experimental systems, as well as population-based observational studies, has led to the recognition of vascular inflammatory processes as central to all stages of atherogenesis, from local endothelial dysfunction to plaque development and rupture [1,2]. Cigarette smoking has been epidemiologically established as a major risk factor for atherosclerosis and shown to promote plaque development in experimental animal models [3-5]. Mechanistically, endothelial dysfunction is thought to be a key initiating cellular event that results from a variety of pro-atherogenic stimuli including cigarette smoke (CS), dyslipidemia and oxidative stress [6-8].

Recent advances in high-throughput technologies have made the analysis of datasets from cardiovascular cells and tissues possible [9]. Current challenges in the analysis of transcriptomics datasets based on functional annotation or pathway maps (e.g. Gene ontology, KEGG) [10,11] reside on the forward reasoning assumption that differential expression of genes is directly related to differential protein activity. The variable relationship of mRNA to protein activity due to post-transcriptional, translational and protein and mRNA degradation regulation [12-14] may lead to misinterpretation of gene expression data. Reverse Causal Reasoning (RCR), a backward computational reasoning methodology, uses observed differential expression of genes in datasets to reverse-formulate mechanistic explanations (termed hypotheses [HYPs]) of the observed effects [15]. RCR uses a large database structure of experimentally-driven causal observations (Selventa Knowledgebase, [SK]) as a substrate for reasoning and HYP generation. Subsequent mapping of HYPs to network models that recreate the biology of interest (e.g., atherogenesis) offers a mechanistically integrated evaluation and interpretation of gene expression data captured in large datasets. Combining prior knowledge from published literature with large “omics” datasets (e.g., transcriptomics) into *in silico* network models accelerates the data interpretation process and our understanding of cellular behavior. Unlike direct network mapping of gene expression data, a network-based HYP evaluation approach allows translating experimentally-determined molecular changes as measurable network perturbations that can be compared between different datasets.

We have previously reported the construction of five network models relating cellular stress, proliferation, DNA damage, autophagy, cell death and senescence, lung inflammation, and tissue repair and angiogenesis in lung and vascular tissues [16-20]. The present work describes the construction and application of the Vascular Inflammatory Processes Network (V-IPN), a network model that combines a molecular framework constructed from publicly available literature and enhanced with RCR data-derived mechanisms, to depict a broad range of inflammatory processes known to occur in vascular tissue during atherosclerotic disease progression. The V-IPN also describes the mechanisms leading to plaque instability, an event in plaque development that often leads to fatal myocardial or cerebral infarction as a result of plaque rupture and vessel occlusion. We used the V-IPN network to assess the degree of biological mechanistic coverage from four different sets of transcriptomics profiling data derived from multiple atherosclerotic-relevant contexts including human endothelial cells (ECs) in culture, coronary arteries from coronary artery disease (CAD) patients and aortas from ApoE^-/-^ mice. The systemic inflammatory status of ApoE^-/-^ mice, a well-established model of atherosclerosis [21], makes this strain an ideal model in which to study comorbidities associated to cigarette smoking [22]. Our results indicate that the V-IPN captures the key biological mechanisms that underlie the progression of vascular disease in various cellular and tissue contexts and allows for a comprehensive interrogation of transcriptomics datasets related to atherogenesis and cross-species translatability.

## Methods

**Figure 1:**
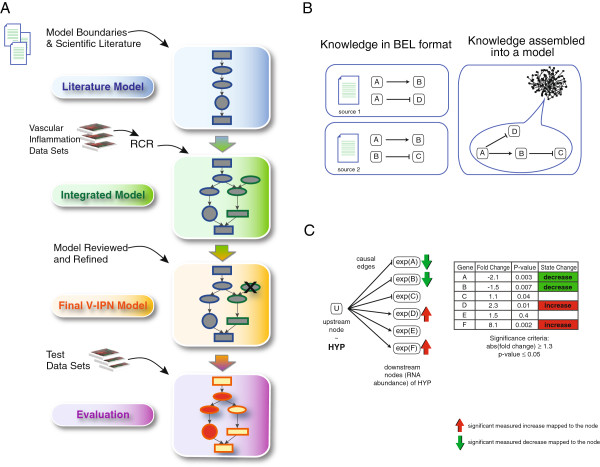
**Workflow diagram showing the model construction used to create the Vascular Inflammatory Processes Network (V-IPN). (A)** The V-IPN is comprised of two primary components: a literature model (blue cylinder) and an RCR-based integration of gene expression vascular datasets (green cylinder). The literature model was constructed from causal connections within well-defined biological contexts and boundaries using published scientific literature. The content of the literature model was augmented with nodes derived from RCR analysis of publicly available vascular inflammation-relevant datasets. The final V-IPN model (orange cylinder) resulted from a comprehensive review of the integrated model. The final model was then evaluated using additional, independent, gene expression datasets to generate HYPs that were mapped to the V-IPN (purple cylinder). **(B)** Assembly of scientific knowledge into a model. Published knowledge is described in Biological Expression Language (BEL) and then integrated and assembled in a model as nodes connected by causal relationships (edges). This assembled model/network serves as a substrate for RCR and HYP generation. Each causal relation in the network is based on one or more scientific findings. **(C)** Mapping of differential measurements to a HYP. A HYP is evaluated as an explanation for the observed changes in gene expression data. Differential measurements in the dataset (RNA abundance of genes A-F expressed as discrete State Changes) are associated with the corresponding downstream nodes of the HYP.

Evaluation of transcriptomics datasets requires executing two sequential processes: RCR-based generation of HYPs and mapping of those HYPs to a network model for evaluation. RCR uses the SK as a substrate for HYP generation, whereas HYP evaluation demands a network model containing the relevant biology. Network construction is a multistep process that also benefits from RCR to augment and refine the literature-based representation of the biology of interest (Figure 1). The sections below describe each element involved in model construction and the RCR-based approach we followed to evaluate transcriptomics datasets obtained from ECs and vascular tissues subjected to atherogenic experimental perturbations.

### Selventa Knowledgebase (SK)

The nodes (biological concepts and entities) and edges (assertions about causal and non-causal relationships between nodes) comprising the V-IPN model were assembled from the SK, a comprehensive repository containing over 1.5 million nodes and over 7.5 million edges. The assertions in the SK are derived primarily from peer-reviewed scientific literature. Each assertion describes an individual experimental observation from a study performed either *in vivo* or *in vitro*. Assertions also capture information about the database source (e.g., PMID for journal articles listed in PubMed), the species (human, mouse or rat) and the tissue or cell type from which the experimental observation was derived. An example of a causal assertion is the increased transcriptional activity of NFkB causing an increase in the mRNA expression of CXCL1 (HeLa cell line; human; PMID 16414985, [23]). The SK contains causal relationships derived from healthy and disease contexts such as inflammation and cardiovascular disease. While the SK is a private commercial resource, a subset of the information contained in it, as well as a freely available implementation of RCR called Whistle, are publicly available (https://github.com/Selventa/whistle). The proportion of vascular specific evidence for network edges as defined by vascular biology keywords (e.g., endothelial cells, smooth muscle cells) is depicted in Table 1 (See Additional file 1: Vascular Biology Keywords).

**Table 1:** Proportion of vascular-specific evidence statements for the V-IPN subnetworks

**Subnetwork**	**Total edges**	**Edges >1 evidence annotated with vascular context**	**Vascular edges/Total (%)**
Endothelial Cell Activation	407	238	58
Smooth Muscle Cell Activation	179	78	44
Plaque Destabilization	494	144	29
Endothelial Cell – Monocyte Interaction	112	27	24
Foam Cell Formation	285	27	9
Platelet Activation	179	10	6

As causal evidence from platelets and inflammatory cells increase in a given subnetwork (e.g. Foam Cell Formation, Platelet Activation), the number of vascular biology-related evidence decreases.

### RCR-based HYP generation process

The RCR methodology utilized for network augmentation has been described previously [15] and a detailed description may be found in the supplementary methods (Additional file 2). Briefly, RCR analysis identifies potential HYPs for the statistically significant mRNA State Changes observed in the transcriptomics datasets. These upstream controllers are termed HYPs, as they represent statistically significant hypotheses that are potential explanations for the observed mRNA State Changes (Figure 1C). Detailed descriptions of the probabilistic scoring metrics (richness and concordance) can be found in Catlett et al. [15], whereas the use of causal assertions in the construction of the V-IPN are further described in the Additional file 2: Supplementary Methods.

### V-IPN construction: model structure and boundaries

The workflow for the creation of the V-IPN is illustrated in Figure 1A. The initial literature-based network scaffold was defined by specific cell, tissue, species and disease contexts (e.g., ECs, aorta, human and atherosclerosis) known to be implicated in vascular pathobiology. The V-IPN nodes and edges comprising the scaffold were assembled in a sequential process by first using causal connections derived from knowledge published in the scientific literature and captured by the SK (Figure 1B).

The literature-derived framework was further augmented with nodes derived from the RCR analysis of vascular inflammation transcriptomics datasets (referred to as “model building” datasets, Table 1). RCR analysis yielded several dozen additional HYPs that were vetted for biological relevance and incorporated into the network as new nodes. Such nodes were connected to the literature scaffold using causal relationships captured by the SK. The resulting integrated network was manually reviewed by scientists with expertise in vascular biology and inflammation. The modular framework consists of six subnetworks that accompany this manuscript in XGMML and .XLS formats (Additional file 3). The network architecture may be viewed from the XGMML files using freely available network visualization software such as Cytoscape (http://www.cytoscape.org/).

### Gene expression datasets used for V-IPN construction and evaluation

Detailed information for each dataset used for model building and evaluation including IDs, self-descriptive names and experimental perturbations is provided in Table 2. Previously published transcriptomics datasets were downloaded from Gene Expression Omnibus (GEO) (http://www.ncbi.nlm.nih.gov/gds). Details of transcriptomics expression analysis of murine aortas (E-MTAB-1696 *[Mm_Ao_16w_ApoE_CS_vs_sham]*) and NHBE cells (E-MTAB-1272 *[Hs_NHBE_CDKinh_rel_vs_blk_8h]*) are provided in the Additional file 2: Supplementary methods. Statistically significant differentially expressed genes were used as input for RCR-based generation of HYPs. Three model-building datasets representing human and murine mechanisms related to vascular pathobiology were utilized for network enhancement (Table 1). Transcriptomics datasets from aortas of 32 and 78 week-old ApoE^-/-^ mice (GSE2372 *[Mm_Ao_32w_ApoE_vs_wt]* and GSE10000 *[Mm_Ao_78w_ApoE_vs_wt]*) were assessed alongside one dataset from primary human aortic endothelial cells (HAECs) exposed to oxidized 1-palmitoyl-2-arachidonoyl-sn-glycero-3-phosphocholine (Ox-PAPC) (GSE29903 *[Hs_EC_oxPAP_vs_PAP]*) to capture a larger spectrum of molecular events characterizing vascular inflammation in both species. The addition of these data-derived HYPs to the literature-based framework generated the integrated model (Figure 1A).

**Table 2:** Datasets analyzed by RCR for V-IPN augmentation and evaluation

	**Dataset name**	**Dataset ID**	**PubMed ID**	**Species**	**Experimental context**	**Tissue/cell type**		**Perturbation**	**Timepoint**	**Independent endpoint**	**Control**
**Model Building Datasets**	*Hs_EC_oxPAP_vs_PAP*	GSE29903	16912112 (Gargalovic, 2006)	Hs	*in vitro*	HAECs		oxPAPC (40 μg/ml)	4 h	IL8 induction	PAPC
											(40 μg/ml)
	*Mm_Ao_32w_ApoE_vs_wt*	GSE2372	19139167 (Grabner, 2009)	Mm	*in vivo*	Aorta		ApoE^-/-^	32 wk of age	Aortic morphometry, IHC, FACS	Wild-type mice (C57BL/6 J)
	*Mm_Ao_78w_ApoE_vs_wt*	GSE10000							78 wk of age		
**Test Datasets**	*Hs_EC_GFP_oxLDL_vs_ct*	GSE13139	19279231 (Mattaliano, 2009)	Hs	*in vitro*	HAECs (cell line)	GFP overexp	oxLDL	24 h	IL8 induction	Untreated
	*Hs_EC_LOX1_oxLDL_vs_ct*						LOX-1 overexp				
	*Hs_EC_oxPAP_vs_ct*	GSE20060	20170901 (Romanoski, 2010)	Hs	*in vitro*	Primary HAECs		Ox-PAPC (40 μg/ml)	4 h	eQTL analysis	Untreated
	*Hs_athCA_vs_ctIMA*	GSE40231	19997623 (Hagg, 2009)	Hs	*in vivo*	Coronary artery		Atherosclerotic lesions	66 ± 8 yr of age	Angiography, blood cytokines	Paired unaffected artery (IMA)
	*Mm_Ao_16w_ApoE_CS_vs_sham*	E-MTAB-1696	-	Mm	*in vivo* (ApoE^-/-^)	Aorta		CS exposure	13-16 wk of age; 30 d exposure	Lipoprotein profile	Fresh air exposure

ECs: endothelial cells; HAECs: human aortic endothelial cells (ECs); GFP: green fluorescent protein; OxPAPC: oxidized 1-palmitoyl-2-arachidonoyl-sn-glycero-3-phosphocholine; oxLDL: oxidized low density lipoprotein; IHS: immunohistochemistry; FACS: fluorescence-activated cell sorting; eQTL: expression quantitative trait loci; Hs: *homo sapiens*; IMA: internal mammary artery; Mm: *mus musculus,* MVECs: microvascular endothelial cells; h: hours; d: days; wk: weeks; yr: years.

Four transcriptomics datasets from isolated human ECs (GSE13139 *[Hs_EC_GFP_oxLDL_vs_ct]*) [24] and atherosclerotic human coronary arteries (GSE40231 *[Hs_athCA_vs_ctIMA]*) [25], as well as murine aortas (E-MTAB-1696 *[Mm_Ao_16w_ApoE_CS_vs_sham]*), were analysed by RCR. RCR results (HYPs) were then used to evaluate network performance by determining HYP-level coverage and odds ratios (OR) across the six subnetworks constituting the V-IPN. Names describing the species and experimental settings for each dataset were created and they were used throughout the results and discussion section to facilitate comparative analyses.

#### Gene expression datasets used as negative controls

Three datasets from normal human bronchial epithelial (NHBE) cells (E-MTAB-1272 *[Hs_NHBE_CDKinh_rel_vs_blk_8h*]), human cardiac and lung microvascular ECs (MVEC-L and MVEC-C) (GSE11341 *[Hs_JurkT_ars_vs_ct]*) [26] and Jurkat cells (GSE23824090 *[Hs_JurkT_ars_vs_ct]*) [27] were used as negative controls (Table 3). NHBE cells and Jurkat cells served as non-cardiovascular controls, whereas lung and cardiac microvascular ECs represent negative control datasets from small vessels, which do not develop atherosclerosis.

**Table 3:** Datasets analyzed by RCR used as negative controls

**Dataset name**	**Dataset ID**	**PubMed ID**		**Species**	**Experimental context**	**Tissue/cell type**	**Perturbation**	**Time point**	**Independent endpoint**	**Control**
*Hs_NHBE_CDKinh_rel_vs_blk_8 h*	E-MTAB-1272	23926424		Hs	*in vitro*	NHBE	Exposure to CDK4/6 inhibitor	24 exposure + 8 h after removal of inhibitor	FACS, cell cycle analysis	Untreated
*Hs_MVEC-C_hpx_vs_ct_24 h*	GSE11341	18469115	Hs	*In vitro*	Primary cardiac microvascular ECs	24 h hypoxia (1% O2)				Normoxia (21% O2)
*Hs_MVEC-L_hpx_vs_ct_24 h*	GSE11341	18469115	Hs	*In vitro*	Primary human pulmonary microvascular ECs	24 h hypoxia (1% O2)				Normoxia (21% O2)
*Hs_JurkT_ars_vs_ct*	GSE46909	23824090	Hs	*In vitro*	Jurkat T cells	Arsenic trioxide 3 μM	6 h exposure			Untreated

*Hs_NHBE_CDKinh_rel_vs_blk_8h, Hs_MVEC-C_hpx_vs_ct_24h, Hs_MVEC-L_hpx_vs_ct_24h* and *Hs_JurkT_ars_vs_ct* were used as a negative control datasets. ECs: endothelial cells; HAECs: human aortic endothelial cells; HCAECs: human coronary artery ECs; GFP: green fluorescent protein; OxPAPC: oxidized 1-palmitoyl-2-arachidonoyl-sn-glycero-3-phosphocholine; oxLDL: oxidized low density lipoprotein; IHS: immunohistochemistry; FACS: fluorescence-activated cell sorting; eQTL: expression quantitative trait loci; Hs: *homo sapiens*; IMA: internal mammary artery; Mm: *mus musculus,* MVECs: microvascular ECs; NHBE: normal human bronchial epithelial cells; h: hours; d: days; wk: weeks; yr: years.

### Calculation of coverage and odds ratio

**Figure 2:**
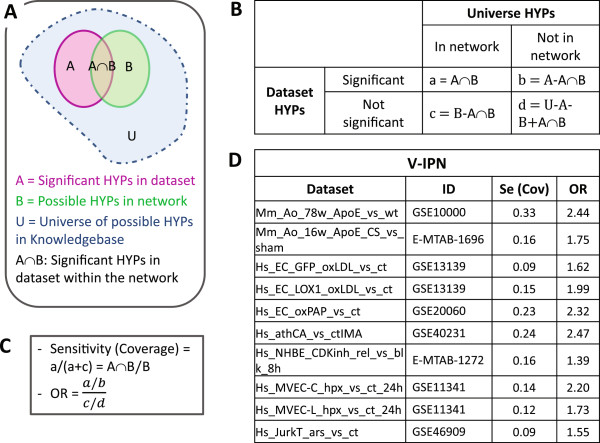
**Calculation of V-IPN coverage and odds ratio (OR). (A)** Schematic representation of the subsets of HYPs in the SK, the network and a given dataset. **(B)** To ascertain the validity of the predictions, two statistical metrics, sensitivity (coverage) and OR, were estimated using HYPs data as depicted in the 2x2 table. **(C)** Coverage is an estimate of the fraction of possible HYPs in a subnetwork that are significant in a given dataset (A∩B/B), whereas the OR is the odds of having significant dataset HYPs in the network (a/b) divided by the odds of having non-significant dataset HYPs (c/d). Coverage is a measure of HYP enrichment within a given subnetwork, whereas OR reflects the probability of having significant dataset HYPs in that subnetwork. An OR higher than 1 implies that the odds of having significant HYPs in a given subnetwork are higher than the odds of having not significant HYPs in that subnetwork. Thus, OR estimates the odds of HYP enrichment. **(D)** Calculated coverage and OR for the entire V-IPN. *Mm_Ao_78w_ApoE_vs_wt* (GSE10000) is included as a building dataset reference. V-IPN: Vascular Inflammatory Process Network, Se: sensitivity, Cov: coverage.

Evaluating the RCR results for each dataset in the context of each of the six V-IPN subnetworks was estimated by calculating coverage and odds ratio (OR). Figure 2 depicts a schematic representation of coverage (sensitivity) and OR, as well as the equations involved in their calculation. The dataset’s coverage was calculated as the fraction of possible HYPs in each subnetwork that are significant HYPs in a given dataset (Figure 2A, B and C). The OR was calculated as the odds of having significant HYPs in the network divided by the odds of having non-significant HYPs in the network (Figure 2B and C). Thus, sensitivity is an estimate of subnetwork coverage (overlap), whereas OR estimates the odds of significant HYP enrichment for a specific dataset-subnetwork pair. An OR higher than one implies that the odds of having significant HYPs in a given subnetwork are higher than the odds of having not significant HYPs in that subnetwork (Figure 2D). The larger the OR of a given dataset, the better the network encompasses the biology embedded in the dataset.

### CS generation and ApoE^-/-^ mice exposure

We set up a study in ApoE-deficient mice in which we investigated the effects of CS on cardiovascular endpoints including plasma lipid profiles, and transcriptomics of aortas. The E-MTAB-1696 *[Mm_Ao_16w_ApoE_CS_vs_sham]* transcriptomics dataset was generated from aortas displaying evidence of atherosclerotic plaques in ApoE^-/-^ mice exposed to cigarette smoke (CS). All animal experimental procedures and CS exposure were approved by an Institutional Animal Care and Use Committee (IUCAC) and are described in detail in the Additional file 2: Supplementary methods. Total cholesterol measurements in plasma, atherosclerotic plaque measurements in the aortic arch and immunohistochemical stainings in vascular tissues of ApoE^-/-^ mice were conducted according to methods detailed in the Addditional file 2: Supplementary methods

## Results

### V-IPN construction and biological integration: description of modular framework and boundaries

**Figure 3:**
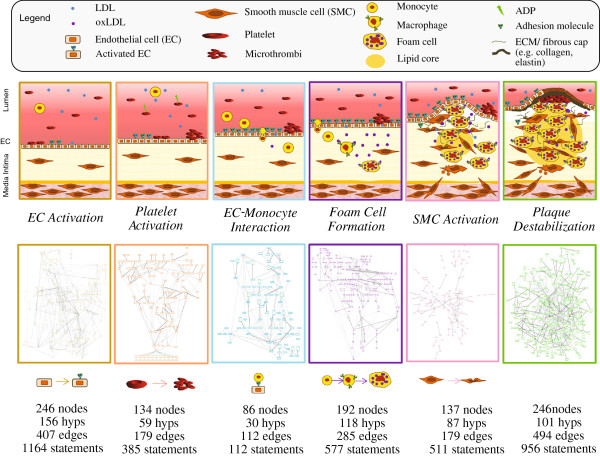
**Biological processes modelled by the V-IPN.** The V-IPN is a modular network model of vascular biology and inflammation. Each subnetwork represents a fundamental set of biological functions, and cellular and molecular players. The modular structure facilitates simulation analysis and evaluation of large ‘omics’ datasets. Five of the six subnetworks describe processes related to the biology of atherogenesis and arterial inflammation; these include *EC Activation*, *Platelet Activation*, *EC-Monocyte Interaction*, *Foam Cell Formation*, and *SMC Activation*. The sixth subnetwork, *Plaque Destabilization*, primarily captures those mechanisms resulting in plaque instability in advanced atherosclerotic disease. The associated subnetwork metrics quantify aspects related to model architecture, such as number of nodes and edges comprising the subnetwork.

To capture the diverse array of biological processes involved in the development of atherosclerotic plaques, the V-IPN network model was constructed using a modular approach that represents key processes related to vascular inflammation and atherogenesis. The biology modelled started with a literature-derived network scaffold followed by a RCR analysis of two murine and one human transcriptomics datasets (“Model Building” Datasets, Table 2) to enhance the representation of biological disease mechanisms from both species. This RCR-based enhancement of the networks helped uncover additional disease-relevant mechanisms not readily identified during the literature-based component of model building. This model building step strengthened the network’s capability to interpret datasets from multiple species. The RCR predictions (HYPs) were included in the network if they had been reported to be mechanistically linked to the process of interest. Biological processes represented in the V-IPN were integrated into six distinct subnetworks that captured key pathobiological events in vascular disease: *Endothelial Cell Activation*, *Endothelial Cell-Monocyte Interaction*, *Foam Cell Formation*, *Platelet Activation*, *Smooth Muscle Cell Activation* and *Plaque Destabilization* (Figure 3). The first five subnetworks describe fundamental atherogenic mechanisms underlying vascular inflammatory responses in discrete cellular populations, whereas the *Plaque Destabilization* subnetwork represents a collection of molecular events that occur in advanced, unstable atherosclerotic lesions. While each subnetwork was constructed to reflect the biological relationships that are involved in specific processes, the subnetworks contain shared elements. For example, since the transcription factor NF-kB is a pleiotropic protein involved in multiple inflammatory pathways, the node “transcriptional activity of NFkB” exists in multiple V-IPN subnetworks, which display varying network connectivity depending on the focus of the subnetwork. Twenty five RCR-predicted HYPs representing mostly pro-inflammatory biological signaling were present in four of the six subnetworks and Ox-LDL was the only HYP common to all subnetworks (Additional file 4: Figure S1). Absolute and relative numbers for all overlapping nodes between subnetworks are shown in Additional file 5: Figure S2. The provided XGMML encoding of the subnetworks allows for the assembly of the V-IPN as a single, agglomerated network using freely available network visualization software such as Cytoscape (http://www.cytoscape.org/).

### Assessment of V-IPN subnetwork-level HYP coverage

#### Test datasets

To test the ability of the V-IPN to identify biological processes modulated by a variety of experimental conditions and models related to atherogenesis, we evaluated a series of *in vitro* and *in vivo* transcriptomics datasets by RCR, the details of which are summarized in Tables 2 and 3. Lists of HYPs for each dataset meeting significance criteria (richness and concordance p-values < 0.05) were evaluated for subnetwork-level coverage, i.e. assessment of HYPs identified as significant in the datasets and also present in the V-IPN. In order to statistically validate the processes being represented in each of the transcriptomics datasets, ORs integrating sensitivity and specificity metrics into a single number were calculated. The overall V-IPN coverage ranged from 9 to 24%, with the human dataset from atherosclerotic coronary arteries *(Hs_athCA_vs_ctIMA)* exhibiting the highest degree of coverage (24%) and the highest OR (2.47) (Figure 2D). Datasets from Jurkat cells *(Hs_JurkT_ars_vs_ct)* and an immortalized EC line *(Hs_EC_GFP_oxLDL_vs_ct)* showed the lowest HYP coverage (9%) (Figure 2D). A summary of the absolute HYP coverage across all six V-IPN subnetworks for each dataset utilized in the network evaluation is presented in Table 4. A significant proportion of transcriptomics data-derived HYPs were captured across the six subnetworks. HYPs predicted from the *Hs_athCA_vs_ctIMA* dataset displayed the highest level of coverage across four V-IPN subnetworks, particularly within the *Plaque Destabilization* subnetwork where coverage approached 40%. The high range of HYP coverage utilizing a dataset derived from human coronary arteries displaying advanced atherosclerotic lesions underscores the validity of the V-IPN in capturing vascular disease-related processes.

**Table 4:** Summary statistics of dataset overlapping across the six V-IPN subnetworks

	**Dataset name**	**Dataset ID**	**N° of state changes (SC)**	**N°of HYPs in dataset**	**N**^**o **^**HYPs overlapping with** ***EC activation***	**N° HYPs overlapping with** ***platelet activation***	**N° HYPs overlapping with** ***EC-monocyte interaction***	**N° HYPs overlapping with** ***foam cell formation***	**N° HYPs O/L with *SMC Activation***	**N° HYPs O/L with *Plaque Destabilization***	**N° HYPs O/L with V-IPN **
**Total N°of possible HYPs in each subnetwork for human/mouse**				2410/ 2354	155/149	59/55	30/27	118/113	88/84	104/102	340*/334
**Mouse**	Mm_Ao_78w_ApoE_vs_wt	GSE10000	3872	449	59	11	13	42	32	43	110
	Mm_Ao_16w_ApoE_CS_vs_sham	E-MTAB-1696	1928	245	24	8	4	23	19	18	52
**Human**	Hs_EC_GFP_oxLDL_vs_ct	GSE13139	398	152	16	4	4	16	9	14	31
	Hs_EC_LOX1_oxLDL_vs_ct	GSE13139	752	224	30	8	8	21	16	21	52
	Hs_EC_oxPAP_vs_ct	GSE20060	609	309	45	12	8	21	23	27	77
	Hs_athCA_vs_ctIMA	GSE40231	3643	309	42	17	11	36	20	37	80
	Hs_NHBE_CDKinh_rel_vs_blk_8h	E-MTAB-1272	1655	296	27	5	7	19	18	14	53
	Hs_MVEC-C_hpx_vs_ct_24h	GSE11341	459	192	27	8	4	16	16	18	48
	Hs_MVEC-L_hpx_vs_ct_24h	GSE11341	504	198	22	4	4	15	13	14	42
	Hs_JurkT_ars_vs_ct	GSE46909	2381	157	15	5	3	3	9	8	31

Reverse causal reasoning (RCR) analysis was conducted using richness and concordance p values <0.05. HYPs that met both criteria were considered to be statistically significant. *Total number of unique human HYPs among the six subnetworks. Total is not 551 HYPs, the sum obtained from Figure 3, due to node/HYP overlap between the subnetworks. *Mm_Ao_78w_ApoE_vs_wt* (GSE10000) is included in this table as a building dataset reference. O/L: overlapping. Dataset Name Dataset ID N° of state changes (SC) N° of HYPs in dataset N° HYPs O/L with *EC Activation* N° HYPs O/L with *Platelet Activation* N° HYPs O/L with *EC-Monocyte Interaction* N° HYPs O/L with *Foam Cell Formation* N° HYPs O/L with *SMC Activation* N° HYPs O/L with *Plaque Destabilization* N° HYPs O/L with V-IPN.

**Figure 4:**
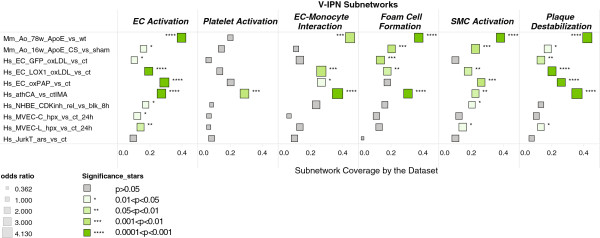
**Dataset coverage (sensitivity) and odds ratio (OR) for significant coverage and enrichment within V-IPN subnetworks.** Subnetwork dataset coverage (sensitivity, see also Figure 2) depicted on the x-axis represents the fraction of possible HYPs in a subnetwork that are significant in a given dataset. Only HYPs meeting both richness and concordance cutoff p-values <0.05 were considered to be statistically significant. OR is an estimate of the probability of having significant dataset HYPs in a given subnetwork. An OR higher than one implies that the odds of having significant HYPs in a given subnetwork are higher than the odds of having not significant HYPs in that subnetwork. ORs were calculated for the overlap between each dataset and each subnetwork. The size of the squares is proportional to the OR. The larger the dataset OR, the better the network encompasses the biology embedded in the dataset. *Hs_NHBE_CDKinh_rel_vs_blk_8h*, *Hs_MVEC-C_hpx_vs_ct_24h* and *Hs_MVEC-L_hpx_vs_ct_24h* were used as negative control datasets. *Mm_Ao_78w_ApoE_vs_wt* is included as a building dataset reference. The significance of HYPs overlap calculated by a chi-square test is depicted by stars. Color intensity indicates significance levels.

Figure 4 depicts the coverage and OR of the overlap for each dataset onto each V-IPN subnetwork. Overall, datasets from immortalized cell lines in culture *(Hs_EC_GFP_oxLDL_vs_ct; Hs_JurkT_ars_vs_ct)* exhibited a lower coverage and OR compared to datasets from intact murine *(Mm_Ao_78w_ApoE_vs_wt; Mm_Ao_16w_ApoE_CS_vs_sham)* and human *(Hs_athCA_vs_ctIMA)* vascular tissues. *(Mm_Ao_78w_ApoE_vs_wt)*, a model construction dataset, is included here only as a reference. Within the EC datasets, primary cells treated with Ox-PAPC (*Hs_EC_oxPAP_vs_ct*) showed the largest coverage compared to both Ox-LDL-treated HAEC datasets *(Hs_EC_GFP_oxLDL_vs_ct; Hs_EC_LOX1_oxLDL_vs_ct)* and MVECs *(Hs_MVEC-C_hpx_vs_ct_24h; Hs_MVEC-L_hpx_vs_ct_24h)*. Within the HAEC study, a larger HYP coverage was shown by LOX-1-transfected ECs *(Hs_EC_LOX1_oxLDL_vs_ct)* compared to cells transfected with GFP *(Hs_EC_GFP_oxLDL_vs_ct)*, suggesting that vascular inflammatory processes are indeed initiated by overexpressing LOX-1.

*Hs_athCA_vs_ctIMA* exhibited statistically significant ORs for all of the six subnetworks and the highest coverage of all datasets (except *Mm_Ao_78w_ApoE_vs_wt*, a model construction dataset) in four of the six subnetworks: *Plaque Destabilization*, *Platelet Activation*, *EC-Monocyte Interaction* and *Foam Cell formation*. This remarkable finding underlines the value of contrasting gene expression datasets from atherosclerotic arteries (e.g. coronary arteries) with normal vessels from the same subjects (e.g., internal mammary arteries) as performed for this dataset [25]. Interestingly, the murine dataset derived from ApoE^-/-^ aortas of old mice *(Mm_Ao_78w_ApoE_vs_wt)* displayed a very similar OR pattern to the human dataset across all subnetworks, with the exception of the *Platelet Activation* subnetwork, which indicates that largely similar biological pathways underlie atherosclerosis in both species (Figure 4). This result also suggests that the process of platelet activation may play a larger role in the development of atherosclerotic plaques in humans compared to advanced-age murine models of atherosclerosis.

#### Negative Control Datasets

Transcriptomics data from four datasets were used as negative controls to evaluate the specificity of the V-IPN. *Hs_NHBE_CDKinh_rel_vs_blk_8h*, a negative control dataset obtained from NHBE cells, exhibited a low degree of coverage across all subnetworks (Figure 4). Significant HYPs for *Hs_NHBE_CDKinh_rel_vs_blk_8h* in the *EC Activation* and *SMC Activation* subnetworks were mostly related to cell cycle and growth factor signaling molecules, which are ubiquitously represented across cell cycle and growth factor subnetworks of the Cell Proliferation Network (Additional file 6: Table S1). Three additional negative control datasets mapped to the V-IPN rendered even lower degrees of coverage compared to NHBE cells (Figure 4). *Hs_MVEC-C_hpx_vs_ct_24h* and *Hs_MVEC-L_hpx_vs_ct_24h*, two datasets obtained from cardiac and lung MVECs subjected to hypoxia, exhibited lower coverage than the dataset from NHBE cells across all subnetworks. Lung MVECs *(Hs_MVEC-L_hpx_vs_ct_24h)* showed a slight degree of coverage in the *EC activation, SMC Activation* and the *Plaque destabilization* subnetworks. *Hs_JurkT_ars_vs_ct*, a control dataset from Jurkat cells, exhibited the lowest degree of coverage of all datasets examined. The low HYP coverage displayed by the negative control datasets from studies using NHBE cells, MVEC and Jurkat cells further demonstrates the specificity of the biology captured by the V-IPN and highlights its value to evaluate processes proximal to vascular immunopathology.

### HYP scoring and HYP directionality

**Figure 5:**
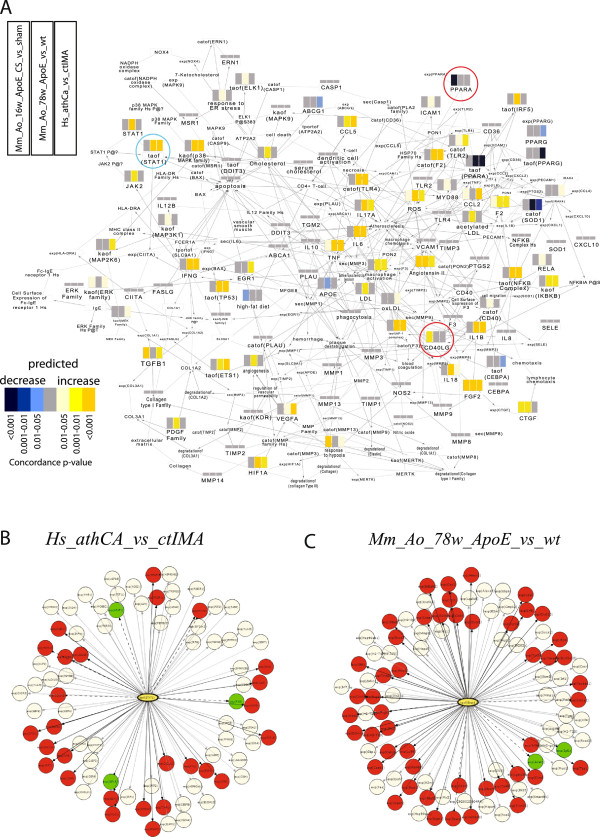
**Plaque destabilization subnetwork coverage and HYP scoring by murine and human datasets. A**. Coverage of the plaque destabilization subnetwork. A chimeric (human/mouse) version of the plaque destabilization network is visualized. Nodes that are possible HYPs have a bar plot indicating if the node is a significant HYP in the *Mm_Ao_16w_ApoE_CS_vs_sham*, the *Mm_Ao_78w_ApoE_vs_wt*, and/or the *Hs_athCA_vs_ctIMA* datasets. HYPs that are predicted down- or up-regulated are blue and orange, respectively. Color intensity reflects statistical significance while grey bars indicate no significant prediction. Bar plots in non-significant nodes for all three datasets were flattened. Circled HYPs in red (PPARA and CD40LG) are two examples of HYPs that were predicted decreased and increased, respectively, in the *Mm_Ao_16w_ApoE_CS_vs_sham* dataset. **B**. HYP scoring of human dataset. Gene expression underlying the HYP with the upstream node taof(STAT1) scored for the *Hs_athCA_vs_ctIMA dataset.***C**. HYP scoring of murine dataset. Gene expression underlying the HYP with the upstream node taof(Stat1) scored for the *Mm_Ao_78w_ApoE_vs_wt* dataset. HYP networks contain measured RNA abundance nodes, represented as circles colored by differential expression (red = significantly increased, green = significantly decreased, white = no significant change). Differentially expressed RNAs mapped to the network includes supporting increased (solid arrows) and decreased (dotted lines) mechanism activity.

The predicted directionality of all significant HYPs for all subnetworks, i.e. increased or decreased predictions based on the downstream gene expression is included in a supplementary file for each dataset examined *(*Additional file 7: *Datasets_Analysis_Dashboards).* A representative mapping of the *Plaque Destabilization* subnetwork depicted in Figure 5A shows bar plots for all possible HYPs that were predicted as significant in the *Mm_Ao_16w_ApoE_CS_vs_sham*, *Mm_Ao_78w_ApoE_vs_wt*, and *Hs_athCA_vs_ctIMA* datasets. This visualization highlights *Hs_athCA_vs_ctIMA* as the dataset exhibiting the largest HYP coverage and enrichment values compared to two murine datasets *(Mm_Ao_16w_ApoE_CS_vs_sham and Mm_Ao_78w_ApoE_vs_wt)*. The subnetwork depiction also shows biological components being largely up-regulated as they relate to the process of plaque destabilization in human disease. A representative example of the gene expression data downstream of HYP *taof(Stat1)* scored for the *Hs_athCA_vs_ctIMA* and the *Mm_Ao_78w_ApoE_vs_wt* datasets is depicted in Figure 5B and C, respectively. The HYP contains 94 and 82 measured RNA abundance nodes and a total of 31 and 50 differentially expressed RNAs mapped to the network from the human and murine datasets, respectively. Twenty-five and 46 genes supporting upregulated activity and 6 and 4 supporting downregulated activity from the human and murine datasets, respectively. The proportion of RNA downstream nodes supporting an increased activity of STAT1 over those supporting a downregulated activity may be related to the key cell signaling role of STAT1 in cells embedded in advanced murine atherosclerotic lesions. Four additional examples of HYP scoring displaying the underlying gene expression data of HYPs for the *Mm_Ao_78w_ApoE_vs_wt* dataset were included in the supplement. Selected HYPs included “macrophage activation”, “Ccl5”, “monocyte adherence” and “kaof(Chuk)” (Additional file 8: Figure S3a-d).

The HYP coverage of *Hs_athCA_vs_ctIMA* was examined not only across all V-IPN subnetworks, but also across all subnetworks and models we have previously published [16-20]. A bar plot visualization of all overlapping networks is depicted in Additional file 9: Figure S4 as coverage (bar length) and OR (grey color intensity). Subnetworks displaying the largest coverage and highest ORs are within the IPN (Inflammatory Process Network), TRAG (Tissue Repair and Angiogenesis) and DACS (DNA damage, Autophagy, Cell death, and Senescence) models and include *Dendritic Cell Migration*, *Neutrophil Chemotaxis, Natural Killer (NK) Cell Activation*, *Epithelial Cell Barrier Defense*, *Macrophage activation*, *Immune Regulation of Angiogenesis* and *MAP kinases (Mapk)*. Many of these subnetworks constitute biological processes that have also been implicated in the development of atherosclerotic lesions.

### V-IPN evaluation of preclinical data translatability

#### V-IPN coverage of predicted HYPs from human *in vitro* datasets

To investigate the ability of the V-IPN to distinguish the effects of different experimental perturbations, we compared the predicted HYPs from three sets of transcriptomics data from HAECs stimulated with Ox-LDL *(Hs_EC_GFP_oxLDL_vs_ct, Hs_EC_LOX1_oxLDL_vs_ct)* or Ox-PAPC *(Hs_EC_oxPAP_vs_ct)*. The largest HYP coverage by a single dataset was observed with *Hs_EC_oxPAP_vs_ct* (40 HYPs, 7-15% across all subnetworks), followed by *Hs_EC_LOX1_oxLDL_vs_ct* (14 HYPs, 2-7%) and *Hs_EC_GFP_oxLDL_vs_ct* (5 HYPs, 0-3%) (Additional file 10: Table S2). Significant HYPs observed to be shared between the three datasets included inflammatory molecules INFB1, IFNG and IL17A. Predicted HYPs known to be transcriptional modulators involved in lipid metabolism of biomembranes (CREB1, SREBF1 and SREBF2) were also commonly observed in the three datasets. Additional significant HYPs in *Hs_EC_oxPAP_vs_ct* reflects a group of growth factors and cell cycle controllers (PDGF, IGF1, CDK4, CCND1, CDKN1A), inflammatory cytokines and chemokines (CCL2, CCL5, CD40LG), oxidative stress-related molecules (NOS3, SOD1), transcriptional regulators of mitogenesis and inflammation (ATF4, NFKB, SP1), and molecules driving cytoplasmic and intra-organelle signaling events leading to migration, proliferation and cell death (AKT, MAPK8, PKA). The results of this coverage analysis suggest that treatment with Ox-PAPC may be a more potent inducer of processes related to atherogenesis when compared to oxLDL treatment, in the specific context of HAECs stimulated *in vitro*.

#### V-IPN coverage of predicted HYPs from human and murine *in vivo* datasets

To test the power of the V-IPN at capturing mechanisms and biological pathways implicated in advanced vascular lesion development in human arteries, we evaluated coverage across the V-IPN subnetworks with a gene expression dataset from human coronary arteries isolated from CAD patients undergoing bypass surgery *(Hs_athCA_vs_ctIMA*) [25]. Lesion stage- and species-specific mechanistic differences were assessed by comparing the HYP coverage of each V-IPN subnetwork by the human dataset with two murine aortic datasets from early *(Mm_Ao_16w_ApoE_CS_vs_sham)* and advanced *(Mm_Ao_78w_ApoE_vs_wt)* atherosclerosis. Aortic tissue was collected from sexually mature 13–16 week-old ApoE^-/-^ mice following 30 days of CS exposure *(Mm_Ao_16w_ApoE_CS_vs_sham)* and from 78 week-old, unexposed ApoE^-/-^ mice *(Mm_Ao_78w_ApoE_vs_wt)*. The *Mm_Ao_78w_ApoE_vs_wt* dataset was used in this evaluation as a reference for stage- and species-specific comparisons with the *Mm_Ao_16w_ApoE_CS_vs_sham* and human *Hs_athCA_vs_ctIMA* datasets, respectively.

V-IPN coverage of HYPs common to the murine and human datasets revealed that more HYPs were common between *Mm_Ao_78w_ApoE_vs_wt* and *Hs_athCA_vs_ctIMA* than between any other dataset pair comparison (*Mm_Ao_16w_ApoE_CS_vs_sham* // *Hs_athCA_vs_ctIMA; Mm_Ao_16w_ApoE_CS_vs_sham* // *Mm_Ao_78w_ApoE_vs_wt)* or the combination of the three datasets. The number of HYPs shared between *Mm_Ao_78w_ApoE_vs_wt* and *Hs_athCA_vs_ctIMA* was 2–5 times higher than any other possible comparison within the three datasets (Table 5). This observation prompted us to conduct murine-murine and murine-human dataset comparisons to further examine the molecules and pathways shared by these datasets. This approach allowed us to evaluate whether distinct species- and lesion stage-specific mechanisms may play a role in vascular lesion development.

**Table 5:** Dataset HYP overlapping between murine and human vascular tissues across the V-IPN subnetworks

***Mm_Ao_16w_ApoE_CS_vs_sham***	***Mm_Ao_78w_ApoE_vs_wt***	***Hs_athCA_vs_ctIMA***	**No of HYPs**	***EC activation***	***Platelet activation***	***EC-monocyte interaction***	***Foam cell formation***	***SMC activation***	***Plaque destabilization***
X	X	X	18	11 (7)	3 (5)	2 (7)	8 (7)	9 (10)	9 (9)
X	X	-	8	2 (1)	0 (0)	0 (0)	4 (3)	3 (3)	1 (1)
X	-	X	6	2 (1)	0 (0)	2 (7)	3 (3)	2 (2)	4 (4)
-	X	X	32	18 (12)	6 (10)	5 (17)	15 (13)	4 (5)	16 (16)
X	-	-	20	9 (6)	5 (8)	0 (0)	8 (7)	5 (6)	4 (4)
-	X	-	51	28 (18)	2 (3)	6 (21)	15 (13)	16 (18)	17 (17)
-	-	X	24	11 (7)	8 (14)	2 (7)	10 (8)	5 (6)	8 (8)
-	-	-	583	73 (47)	36 (61)	12 (41)	55 (47)	43 (49)	44 (43)

Numbers between parentheses are percentage of total number of possible HYPs.

##### Early vs advanced murine atherosclerosis datasets

**Figure 6:**
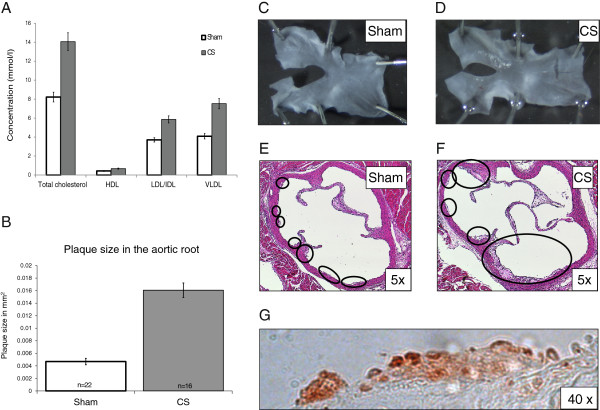
**Atherogenesis in ApoE-/- model after 30 days of CS exposure. (A)**. Concentration of lipoproteins in serum of ApoE^-/-^ mice exposed to sham or to CS for 30 days. **(B)**. Plaque area in the aortic arch of sham and CS-exposed mice. **(C/D)**. Representative images of the aortic arch of 12 week old Apo E^-/-^ mice after the end of the exposure period. **(E/F)**. Representative images of H&E stained cross sections of the aortic roots of Apo E^-/-^ mice at the end of the exposure period. Atherosclerotic lesions are encircled. **(G)**. Immunohistochemical staining with an anti-Mac-3 antibody revealed that atherosclerotic lesions from the aortic root of a CS-exposed animal displayed significant presence of macrophages.

The predicted HYPs from the murine datasets showed a low degree of overlapping HYP coverage (0-3%, Table 5), suggesting that distinct molecular pathways are active at various stages of atherogenesis in the same species. The *Mm_Ao_16w_ApoE_CS_vs_sham* dataset alone covered 0-7%. We sought to evaluate the coverage of this dataset across the V-IPN subnetworks to determine the mechanistic similarities and differences underlying lesion formation following CS-exposure when compared to advanced-age lesions in the same species. To determine the extent of vascular lesion development in the CS-exposure model, aortic histomorphometry and plasma lipid profiles were performed at the end of the study. Total cholesterol, LDL and VLDL from animals exposed to CS for 30 days were significantly increased, as was the size of atherosclerotic plaques in the aortic arch (Figure 6, panels A to D). H&E staining of cross-sections of the aortic roots showed typical intimal thickenings in areas close to the aortic valve leaflets; immunohistochemical staining of these lesions with a MAC3 antibody indicated that they were infiltrated by numerous macrophages (Figure 6, panels E to G).

Predicted HYPs that were common to the mouse datasets from young and old mice *(Mm_Ao_16w_ApoE_CS_vs_sham* and *Mm_Ao_78w_ApoE_vs_wt)* indicated a low degree of overlapping across the V-IPN subnetworks (0-3%, Table 5). Coverage analysis showed some mechanisms and molecules being activated solely in the *Mm_Ao_16w_ApoE_CS_vs_sham* dataset including PPARA, CD40LG, RAC1, PGE2 and SREBF2. Two predicted HYPs were found in four out of the six subnetworks (decreased PPARA and increased CD40LG) including the *Plaque Destabilization* subnetwork (Figure 5). AGTR1A (angiotensin II receptor 1A) was also a predicted HYP shared by both datasets in three subnetworks. These results indicate that in addition to a small set of mechanisms shared by early and advanced murine lesions, distinct biological pathways may also contribute to lesion formation in CS exposure-induced early vascular lesions compared to those implicated in advanced, older lesions.

##### Advanced murine vs. advanced human atherosclerosis datasets

In contrast to the murine dataset comparison results, the degree of overlapping HYPs between *Mm_Ao_78w_ApoE_vs_wt* and *Hs_athCA_vs_ctIMA* ranged from 10-16% (Table 5), except for the *SMC Activation* subnetwork (5%). Many significant HYPs were shared between these two datasets within the *Plaque Destabilization* subnetwork, (Figure 5A). Significant HYPs related to vascular pathobiology were mapped to the V-IPN; they included the processes of inflammation, angiogenesis, and monocyte and macrophage differentiation. Common HYPs included the canonical transcriptional regulators AP1, IRF3, REL and SPI1, nuclear receptors and signal transducers (e.g., NCOR1, STAT1), growth factors (e.g., VEGFA), and chemoattractants (e.g., CCL5). Cytokines involved in the differentiation and function of macrophages and lymphocytes CSF1, CSF2, IFNG, IL1B and IL6, were also shared between the two datasets. A gene functional clustering analysis of common HYPs using DAVID (http://david.abcc.ncifcrf.gov/home.jsp) revealed inflammation, cytokine activity, chemotaxis and the toll-like receptor signaling pathways as the top ranked functional categories (Additional file 11: Table S3). This HYP coverage analysis indicates that a shared repertoire of biological mechanisms underlies the development of advanced vascular lesions in both humans and mice.

##### Early murine vs. advanced human atherosclerosis

A comparison between *Mm_Ao_16w_ApoE_CS_vs_sham* and *Hs_athCA_vs_ctIMA* revealed a low degree of overlapping HYPs, ranging from 0-7% spanning all subnetworks (Table 5). Furthermore, coverage analysis within the *Plaque Destabilization* subnetwork indicated only a few HYPs being shared between these two datasets (increased CCL2, increased “response to hypoxia”, and increased TGFB1) (Figure 5A). Only two additional HYPs, HIF1A and PPARD, were shared by both datasets in other subnetworks. This analysis suggests that despite a comparable atherosclerotic plaque morphology, the molecular events leading to its development in a CS exposure murine model are quite distinct from the molecular pathways leading to plaque formation and destabilization in advanced human lesions.

#### V-IPN-generated functional causal paths in atherosclerotic coronary arteries of CAD patients

*Hs_athCA_vs_ctIMA* dataset coverage analysis showed *Platelet Activation, Plaque Destabilization, Foam Cell Formation* and *EC-Monocyte Interaction* as the V-IPN subnetworks with the highest HYP coverage (Figure 4). In contrast, *SMC Activation* exhibited the lowest coverage. V-IPN mapping of significant HYPs reflected a rich set of biological functions and molecules potentially involved in the pathophysiology of advanced, unstable, atherosclerotic lesions. Some of the most relevant groups are delineated below.

##### Lipid metabolism

A series of significant HYPs related to lipid metabolism were captured by the V-IPN from *Hs_athCA_vs_ctIMA*. They included ABCG1, LDLR, Ox-LDL and Ox-HDL.

##### Platelet function and angiogenesis

Platelet-related HYPs included the thrombin receptor (F2R) and angiopoietin 1 (ANGPT1). F2R is involved in the regulation of the thrombotic response, whereas platelet release of ANGPT1 following platelet activation may be related to a role of platelets in maintaining vascular stability [28]. ANGPT1 has roles in vascular development and may be involved in neovascularization within the plaque.

##### Intracellular signaling

A number of signal transduction molecules including MAP kinases (MAPK1, MAPK3, MAPK8, MAP2K4, MAP2K6, MAP3K5), PI3K as well as transcription factors (CEBPA, EGR1, GATA6) were all predicted as significant HYPs, confirming that multiple signaling pathways account for the behavior of the various cell types present in the diseased atherosclerotic milieu.

## Discussion

### Pathobiological content of the V-IPN

Early mechanisms in vascular disease development such as arterial cell dysfunction are amenable to controlled experimental perturbations in animal models or *in vitro* settings. In contrast, advanced atherosclerotic lesions are challenging to recreate experimentally, which has led to a paucity of sound data on the precise cellular and molecular mechanisms leading to plaque instability and eventual rupture. Disease modelling approaches, such as the implementation of the V-IPN reported here, overcome these barriers by integrating current knowledge and large gene expression datasets into networks reflecting the pathobiology of interest. Each new dataset mapping on the network has the potential to expand our mechanistic knowledge and further refine the network’s structure and content.

Impaired endothelial production of prostacyclin (PGI2) and nitric oxide (NO) in early arterial lesions facilitate vasoconstriction, inflammation and oxidative stress at a time when no morphological changes in the vessel wall have occurred [29]. Cell adhesion molecules (CAMs) are well represented in the V-IPN as they have been extensively documented in the migration of inflammatory cells from the vascular lumen to the subendothelial space [30]. In the presence of hypercholesterolemia and a local excess of reactive oxygen species, oxidation of lipids and lipoproteins leads to activation of phagocytic cells that set in motion a cascade of inflammatory events including the release of growth and chemotactic factors as well as the migration and proliferation of SMCs [31]. Excess lipid uptake by macrophages promotes their differentiation into foam cells, which is the hallmark of fatty streaks observed in early atherosclerosis. We captured the signaling mechanisms responsible for these phenomena in the *SMC Activation* and *Foam Cell Formation* subnetworks, respectively. In humans, the final stage of atheroma formation is reached after years of continuous exposure to environmental noxious stimuli, such as cigarette smoke and a diet rich in saturated fats and poor in antioxidants [29,32]. The natural disease progression results in plaque growth and positive vessel remodeling to maintain a functional lumen size. Although atherosclerotic plaques remain clinically silent for decades, they may evolve to become advanced lesions that are prone to calcification, cap thinning, hemorrhage and rupture. Platelets play a major role in the advanced stages of plaque development where neovascularization, thrombosis, plaque erosion and rupture constitute fatal complications [33]. Interestingly, platelets also participate in early atherogenic events by promoting EC activation and forming microthrombi in fatty streaks [34]. These platelet-related pathways have been captured in the *Plaque Destabilization* and *Platelet Activation* subnetworks. Thus, the pathobiology incorporated in the V-IPN represents a comprehensive implementation of our current knowledge of atherogenesis, which includes the primary cellular players, as well as the array of biological processes involved, ranging from EC dysfunction to the formation of fatty streaks, atheromas, and subsequent plaque instability.

Comparisons of RCR-based analyses of transcriptomics datasets from cells in culture and intact murine and human tissues rendered a series of powerful insights. The underlying molecular findings as well as the pathobiological relevance are described in detail below and summarized in Additional file 12: Table S4.

### The V-IPN captured predicted HYPs from primary HAECs and an immortalized HAEC line that may account for the divergent phenotypes exhibited by the two cell types

ECs uptake oxidized lipids and Ox-LDL through the scavenger receptor LOX-1 [8]. Oxidized lipids induce inflammatory responses mediated by NFkB, including upregulation of cytokines, chemokines and CAMs that result in substantial leukocyte recruitment [35,36]. Predicted HYPs from HAECs overexpressing LOX-1 or GFP *(Hs_EC_LOX1_oxLDL_vs_ct; Hs_EC_GFP_oxLDL_vs_ct)* differed significantly from the HYP profile of HAECs stimulated by proinflammatory oxidized phospholipid, Ox-PAPC *(Hs_EC_oxPAP_vs_ct)*. A distinction between the three datasets is indicated by the relatively low number of predicted HYPs observed in Ox-LDL-treated HAECs; 5 and 14 HYPs for GFP- and LOX-1-transfected cells, respectively, compared to 40 significant HYPs predicted from *Hs_EC_oxPAP_vs_ct*, a dataset generated from HAECs treated with Ox-PAPC (Additional file 10: Table S2). The marked differences in the number of significant predicted HYPs from each dataset could reflect differing magnitudes of signaling events elicited upon stimulating ECs with atherogenic lipids. Indeed, Ox-PAPC, a purified component of Ox-LDL, may have wider and more potent effects on EC biology compared to Ox-LDL [37]. The analysis presented herein could indicate that Ox-PAPC treatment is a more efficient process by which to induce the molecular features that most closely resemble chronic development of atherosclerotic plaques. Alternatively, the differences could be explained by the phenotypic differences between the cell types used and the number of human donors represented in each dataset. Cells from dataset *Hs_EC_LOX1_oxLDL_vs_ct* were obtained from an immortalized (SV40-induced) human aortic EC line [38], whereas primary HAEC cultures from 96 donors were used to generate *Hs_EC_oxPAP_vs_ct* expression data [39]. Cellular transformation of immortalized cell lines is associated with phenotypic changes at multiple levels including gene expression, biochemical, metabolic and proliferative capacity [40,41]. Therefore, transformed cell lines may have more limited abilities to respond to experimental induction by atherogenic lipids.

### Mechanisms identified by the V-IPN discriminate between early and late vascular lesions in ApoE^-/-^ mice and highlight the commonalities between advanced murine and human atherosclerotic lesions

We have conducted RCR analysis on gene expression datasets from a study utilizing the ApoE^-/-^ mouse strain, a well-established model of atherosclerosis [42]. The minimal overlap of common HYPs (6 total, Table 5) between aortas of young *(Mm_Ao_16w_ApoE_CS_vs_sham)* and old *(Mm_Ao_78w_ApoE_vs_wt)* adult ApoE^-/-^ mice demonstrates a substantial divergence of atherogenic processes taking place in young ApoE^-/-^ mice (16-week old) exposed to CS for 30 days compared to advanced atherosclerotic disease observed in older ApoE^-/-^ mice (78 weeks). Strikingly, predicted HYPs from human *(Hs_athCA_vs_ctIMA)* and old murine *(Mm_Ao_78w_ApoE_vs_wt)* datasets generated from intact arteries harboring advanced atherosclerotic lesions shared a significantly larger set of causal mechanisms (32 total HYPs, Table 5) indicating that similar mechanisms underlie the development of advanced atherosclerotic lesions in both species. The pathobiological picture that emerged when overlaying predicted HYPs from both datasets onto the V-IPN is one where a complex series of proliferative, apoptotic and inflammatory events driven by intracellular transducers and transcription regulators are all taking place simultaneously. A functional clustering analysis of the common HYPs from both species using DAVID revealed functional categories consistent with the mechanisms described above (Additional file 11: Table S3). The vast majority of this set of commonly mapped HYPs, which were predicted increased in the human *(Hs_athCA_vs_ctIMA)* and old murine *(Mm_Ao_78w_ApoE_vs_wt)* datasets, should serve as an initial reference for future simulations using murine and human vascular datasets. Our results highlight the power of the V-IPN to assess, at the molecular level, the degree of translatability from murine morphologic [43] and tomographic data [44] on plaque instability [45] to the human clinical setting using transcriptomics data.

### V-IPN evaluation reveals that distinct molecular pathways contribute to atherogenesis in a CS-exposure murine model of disease

Among the predicted HYPs from the mouse *Mm_Ao_16w_ApoE_CS_vs_sham* and *Mm_Ao_78w_ApoE_vs_wt* datasets, coverage analysis across the V-IPN revealed a remarkably low degree of overlapping mechanisms across the V-IPN subnetworks (0-3%, Table 5). This result suggests that distinct biological pathways contribute to early lesion development in ApoE^-/-^ mice exposed to CS. A comparison between *Mm_Ao_16w_ApoE_CS_vs_sham* and *Hs_athCA_vs_ctIMA* also revealed a low extent of overlapping HYPs, ranging from 0-7% spanning all subnetworks (Table 5). Furthermore, broad coverage analysis of the predicted HYPs indicated that the *Mm_Ao_16w_ApoE_CS_vs_sham* dataset contained 52 HYPs found across the V-IPN, whereas the *Hs_athCA_vs_ctIMA* dataset contained 80 HYPs (Table 4). This result suggests that a more diverse array of biological mechanisms underlie advanced atherosclerotic lesion development in the human disease. Indeed, coverage analysis specifically within the *Plaque Destabilization* subnetwork indicated only a few unique mechanisms being activated in the *Mm_Ao_16w_ApoE_CS_vs_sham* samples (e.g. decreased PPARA and increased CD40LG) (Figure 5). A full characterization of discrete cellular events distinguishing acute atherogenesis in a murine model from advanced-stage murine and human disease further demonstrates the utility of the V-IPN in evaluating novel datasets to better understand comparability between species and/or disease model systems.

### V-IPN-generated causal paths were consistent with advanced, unstable lesions, in coronary atherosclerotic arteries of CAD patients

Unlike all other datasets used for model construction and evaluation, *Hs_athCA_vs_ctIMA* represents paired gene expression profiles from 37 human atherosclerotic coronary arteries and control internal mammary arteries (IMA); each pair was obtained from the same subject (STAGE study) [25]. A principal component analysis (PCA) of the gene expression profiles identified two groups of control and diseased arteries (Additional file 13: Figure S5). All pairs were clearly clustered, thus providing a sense of how distinct the gene profile of advanced coronary atherosclerosis was, compared to the reference IMA. Pathological evidence indicates that unstable plaques are complex lesions with common morphological features including a thin fibrous cap, a large lipid core, a network of vasa vasorum and focal inflammation [46]. HYP coverage analysis of the human dataset *(Hs_athCA_vs_ctIMA)* was distinctly consistent with mechanisms driving the morphological features of unstable plaques. Remarkably, the *Plaque Destabilization* and *Platelet Activation* subnetworks exhibited the largest ORs for HYP overlap across all datasets examined and through the six V-IPN subnetworks (Figure 4). The ORs for the *Hs_athCA_vs_ctIMA* dataset were even higher than those obtained for *Mm_Ao_78w_ApoE_vs_wt;* in the *Platelet Activation* (2.9 vs. 1.1) and *Plaque Destabilization* subnetworks (4.1 vs. 3.4); *Mm_Ao_78w_ApoE_vs_wt* is a dataset from advanced murine aortic lesions used for network construction. This analysis highlights model-level coverage of additional mechanisms unique to the human condition and validates the strength of the V-IPN to differentiate expression data derived from advanced human and murine lesions. Consistent with a decreased activity of SMCs leading to fibrous cap thinning in lesions that are prone to rupture, the *SMC Activation* network exhibited lower coverage and ORs compared to both murine datasets, *Mm_Ao_16w_ApoE_CS_vs_sham* and *Mm_Ao_78w_ApoE_vs_wt* (2.1 vs. 2.7 and 2.8) (Figure 4). Significant HYPs categorized within pathobiological functions linked to plaque destabilization are described below.

#### Lipid metabolism-related HYPs

Lipid metabolism-related predicted HYPs included ABCG1, an ATP-binding cassette transporter that regulates macrophage cholesterol efflux and phospholipid transport to lipoprotein acceptors. Intact endothelium from ABCG1-deficient mice has been shown to exhibit 4-fold increases in monocyte adhesion [47]. Other lipid metabolism HYPs included the low density lipoprotein receptor (LDLR), a protein expressed by human macrophages and known to be recycled between the plasma membrane and lysosomes upon binding of LDL. Ox-LDL and Ox-HDL were both highlighted as predicted HYPs. Relaxing the concordance and richness p values from 0.05 to 0.1 resulted in a few additional predicted HYPs relevant to atherogenesis including SP1, CD36 and NOS3. S1P receptor is expressed by ECs and it binds its ligand S1P, a bioactive lipid with numerous functions in the immune and cardiovascular systems. Using a lipidomics approach, we have previously shown that CS exposure increases, whereas cessation decreases, the levels of S1P in plasma of ApoE^-/-^ mice [5,22]. Furthermore, CD36 is a glycoprotein expressed in various vascular and circulatory cell types including monocytes, macrophages, platelets, ECs and adipocytes. It binds collagen, thrombospondin, phospholipids, and Ox-LDL. In macrophages of human atherosclerotic lesions, CD36 acts as a receptor for Ox-LDL and a transporter of long-chain fatty acids. CD36-deficient patients were shown to have hypertriglyceridemia [48], whereas patients with acute coronary syndrome exhibited 6-fold higher levels of CD36 in circulating monocytes compared to healthy controls [49]. Taken together, these data demonstrate that the scavenger receptor CD36 is involved not only in pro-atherogenic mechanisms but also in the development of acute coronary syndrome symptoms, which are primarily caused by plaque rupture at sites of thrombus formation.

#### HYPs related to platelet activation and clot formation

The human advanced coronary lesion dataset *(Hs_athCA_vs_ctIMA)* displayed a high degree of coverage and enrichment within the *Platelet Activation* subnetwork, highlighting the involvement of platelets in thrombus formation and plaque instability. CD36 expression on the surface of platelets serves as an adhesion molecule and a receptor for thrombospondin and Ox-LDL, which was also a predicted HYP mapped to the V-IPN. CD36 has been demonstrated to play a role in platelet activation and thrombus formation in experiments where immobilized thrombospondin and Ox-LDL activate platelets via CD36 through a Syk kinase-dependent signaling mechanism [50]. In agreement with the multiple functions of CD36, the CD36 node is present in three V-IPN subnetworks, *Platelet Activation*, *EC-Monocyte Interaction* and *Plaque Destabilization*; CD36 was a predicted HYP in both the murine and human dataset. A predicted HYP unique to the human dataset was the thrombin receptor. The first step of the coagulation cascade involves cleavage of coagulation factor II to form thrombin. Protease-activated receptors (PARs [FR2]) are activated in response to TF-VIIa-Xa, a ternary complex that is also linked to inflammation within plaques prone to rupture [51]. Plaque vulnerability has been shown to be correlated with PAR1 expression in ApoE^-/-^ mice [52].

#### Angiogenesis-related causal paths were captured by the V-IPN

Other significant HYPs predicted by the datasets included NOS3 and ANGPT1, both of which regulate vascular tone and permeability, as well as blood vessel maturation and stability. Intra-plaque neovascularization may lead to hemorrhage, fissure development and plaque rupture. The neovascularization process that occurs in advanced lesions [53] indicates that various pro-angiogenic factors are being secreted within the plaque and that signaling pathways and associated molecules are all operating in advanced atherosclerotic lesions. In addition to ANGPT1, vascular endothelial growth factor A (VEGFA), HIF1A, a master regulator of a cellular homeostatic response to hypoxia that activates transcription of genes involved in angiogenesis, PTAFR and STAB1, were also identified as significant HYPs and potentially modulated mechanisms. STAB1, also known as CLEVER-1, is a glycoprotein involved in scavenging, angiogenesis and cell adhesion, and has also been shown to mediate transmigration of leukocytes [54].

### Model limitations

RCR-based models do not operate with integrated feedbacks and non-linear elements that contribute to regulating a dynamic output. In order to make accurate predictions, mathematical models incorporate feedback elements tuned to match phenotypic constrains (e.g., blood pressure values). In RCR, all biological feedbacks are implicitly integrated in the datasets. RCR-based models do not dynamically model the regulatory processes controlling biological pathways. RCR-based models are tools to extract biological processes embedded in large sets of molecular data driven by specific experimental perturbations, and to contextualize those findings within a body of knowledge.

## Conclusion

In summary, we have demonstrated that RCR analysis of large gene expression datasets coupled with HYP mapping to the V-IPN was able to discern the mechanistic variability underpinning the development of atherosclerotic lesions in a variety of experimental and species contexts. The mapping of predicted HYPs to the V-IPN was able to successfully distinguish between early and advanced murine lesions, as well as advanced murine and human atherosclerosis, thus pointing to a distinct subset of mechanisms that are translatable to the human condition. Importantly, our computational model proved to be a powerful tool to further our pathophysiological understanding of vascular inflammation, atherogenesis and plaque destabilization. The dynamic nature of the model’s structure allows for further refinement as additional datasets become available and represents a useful tool for the interrogation of cross-species translatability in the context of cardiovascular disease.

### Glossary

Reverse Causal Reasoning (RCR): A computational methodology for identifying potential upstream controllers leading to differential molecular profiles.

Selventa Knowledgebase (SK): A network representing a working set of knowledge fit for a specified use. The SK is used as a substrate for RCR. It encodes prior scientific knowledge as a network of nodes that are connected by edges.

Biological Expression Language (BEL): The knowledge representation language used to build the SK.

Node: A biological entity or process in the SK.

Edge A causal relationship (e.g., increase, decrease, subset) connecting two nodes in the knowledgebase.

State Change (SC): A differential measurement across a sample group (e.g., treated and control) that is converted to a discrete value of increase, decrease, or no change, based on two statistical metrics: richness and concordance.

Hypothesis (HYP): A small, directed causal network containing an upstream node representing a biological entity or process connected by a causal increase, decrease or ambiguous edges to downstream nodes representing measured entities.

HYP upstream node: A controller of downstream nodes in a HYP and a potential explanation for state changes (SC) mapped to the downstream nodes.

HYP downstream nodes: Nodes in a HYP mapped to quantities measured in the dataset.

HYP causal edges: The causal relationships (i.e., increases or decreases) connecting the HYP upstream node to each downstream node.

Richness: A measure of the relevance of a HYP to the changes observed in an experimental dataset.

Concordance: A measure of the consistency of the direction of the changes observed in an experimental dataset.

Coverage (sensitivity): An estimate of the fraction of possible HYPs in a subnetwork that are significant in a dataset. Coverage is a measure of HYP enrichment.

Odds ratio (OR): The probability of having significant dataset HYPs within a network. The higher the OR, the better the network encompasses the biology embedded in a given dataset.

X: Protein abundance of X

taof(X): Transcriptional activity of X

exp(X): RNA expression of X

gtpof(X): GTP-bound activity of X

kaof(X): Kinase activity of X

paof(X): Phosphatase activity of X

catof(X): Catalytic activity of X

X P@Y: Abundance of X phosphorylated at Y

## Abbreviations

BEL: Biological expression language; HYP: Hypothesis; OR: Odds ratio; RCR: Reverse causal reasoning; SC: State change; SK: Selventa knowledgebase.

## Competing interests

The authors declare no competing interests. Héctor De León, Stéphanie Boué, Walter K Schlage, Stephan Gebel, Marja Talikka, Emilija Veljkovic, Michael J Peck, Carole Mathis, Carine Poussin, Katrin Stolle, Julia Hoeng, Manuel C Peitsch are employees of Philip Morris International R&D. Natalia Boukharov, Jurjen W Westra, Aaron VanHooser, R Brett Fields, Vy Hoang, and Renee Deehan are employees of Selventa.

## Authors’ contributions

HDL, SB and MT contributed to dataset model mapping and data interpretation. RBF and NB contributed to data interpretation. HDL, SB and WKS participated in data processing and analysis, and in data interpretation. HDL, RBF and NB drafted and revised the manuscript. SB, WKS, NB, SG, MT, EV, MJP, CM, CP, and KS participated in model conception and design, and in literature curation and vetting. JWW, AVH, VH, and RD participated in model conception and design, and in network construction. JH and MCP contributed to model conception and design. NB and SG participated in network construction. SB, WKS, NB, JH, and MCP revised the manuscript. All authors read and approved the final manuscript.

## Supplementary Material

Additional file 1 Vascular Biology Keywords. Click here for file

Additional file 2 Supplementary Methods. Click here for file

Additional file 3 **Six .XGMML and six .XLS files of all V-IPN subnetworks.** The network architecture may be viewed from the XGMML files using freely available network visualization software such as Cytoscape (http://www.cytoscape.org/). Click here for file

Additional file 4: Figure S1 Frequency rate of nodes and HYPs across the six subnetworks. As the number of events increases, the frequency of those occurrences in all networks decreases. Twenty five HYPs were present in all subnetworks at a simultaneous event rate of 4, whereas a single HYP, Ox-LDL, was present once in all networks. Click here for file

Additional file 5: Figure S2 Node overlap between subnetworks. Table A shows the number of overlapping nodes between all six of the individual subnetworks. Table B shows, as a percentage, the degree of node overlap between the six subnetworks. Colored cells reflect the degree of overlap from low (dark blue) to high degrees of overlap (dark red). Click here for file

Additional file 6: Table S1 Common HYP coverage of V-IPN and cell proliferation subnetworks by a dataset from NHBE cells *(Hs_NHBE_CDKinh_rel_vs_blk_8h)* used as a negative control. (↑) predicted increased, (↓) predicted decreased. Click here for file

Additional file 7 Datasets_Analysis_Dashboards. Click here for file

Additional file 8: Figure S3 **A**. The HYP with the upstream node macrophage activation scored for the *Mm_Ao_78w_ApoE_vs_wt* dataset. This HYP contains 23 measured RNA abundance nodes, represented as circles colored by differential expression (red = significantly increased, green = significantly decreased, white = no significant change). A total of 18 differentially expressed RNAs mapped to the HYP network, including 15 supporting increased mechanism activity (solid arrows) and three supporting decreased activity (dotted lines). **B**. The HYP with the upstream node Ccl5 scored for the *Mm_Ao_78w_ApoE_vs_wt dataset*. This HYP contains 41 measured RNA abundance nodes, represented as circles colored by differential expression (red = significantly increased, green = significantly decreased, white = no significant change). A total of 24 differentially expressed RNAs mapped to the HYP network, including 19 supporting increased mechanism activity (solid arrows) and five supporting decreased activity (dotted lines). **C**. The HYP with the upstream node monocyte adherence, scored for the *Mm_Ao_78w_ApoE_vs_wt dataset*. This HYP contains 87 measured RNA abundance nodes, represented as circles colored by differential expression (red = significantly increased, green = significantly decreased, white = no significant change). A total of 36 differentially expressed RNAs mapped to the HYP network, including 33 supporting increased mechanism activity (solid arrows) and three supporting decreased activity (dotted lines). **D**. The HYP with the upstream node kaof(Chuk), scored for the *Mm_Ao_78w_ApoE_vs_wt dataset*. This HYP contains 44 measured RNA abundance nodes, represented as circles colored by differential expression (red = significantly increased, green = significantly decreased, white = no significant change). A total of 25 differentially expressed RNAs mapped to the HYP network, including 23 supporting increased mechanism activity (solid arrows) and two supporting decreased activity (dotted lines). Click here for file

Additional file 9: Figure S4 Coverage and OR of the dataset *Hs_athCA_vs_ctIMA* (GSE40231) across other network models. Subnetworks with less than 10 HYPs were not included. IPN: Inflammatory Process Network, TRAG: Tissue Repair and Angiogenesis. DACS: DNA damage, Autophagy, Cell death (apoptosis and necroptosis), and Senescence. Click here for file

Additional file 10: Table S2 Transcriptomics-based evaluation of the effects of oxidative stimuli on primary HAEC cultures vs. immortalized HAECs. Click here for file

Additional file 11: Table S3 DAVID functional clustering of common HYPs between *Mm_Ao_78w_ApoE_vs_wt* and *Hs_athCA_vs_ctIMA.* (↑) predicted increased in both datasets; (↓) predicted decreased in both datasets. Click here for file

Additional file 12: Table S4 Summary of findings and insights provided by each dataset evaluated by RCR and the V-IPN. Click here for file

Additional file 13: Figure S5 Principal component analysis (PCA) of samples from *Hs_athCA_vs_ctIMA* (GSE40231). PCA plot A illustrates the principal components of the gene expression profiles of 37 pairs of samples from the atherosclerotic coronary arteries and control internal mammary arteries from the STAGE study. Although the separation between atherosclerotic tissue and control mammary artery is relatively clear, the pairing of the samples (each pair from one patient) empowers the downstream analysis as illustrated when looking at the distance between the pairs of samples. PCA plot B highlights the relationships between the paired samples, demonstrating that for the samples that may look at the borderline between the two groups (ATHERO and CTRL), the difference between the atherosclerotic vessel and its control artery is still very clear, and in the same direction as for the other pairs. Click here for file
