# Supplementary material for: A vascular biology network model focused on inflammatory processes to investigate atherogenesis and plaque instability
Source: J Transl Med. 2014 Jun 26;12:185. doi: 10.1186/1479-5876-12-185 (PMC4227037; doi:10.1186/1479-5876-12-185)
Supplement: Additional file 2 — Supplementary Methods. [file 1479-5876-12-185-S2.docx]

**A vascular biology network model focused on inflammatory processes to investigate atherogenesis and plaque instability**

Héctor De León^1*+^
^*^ Corresponding author
Email: hector.deleon@pmi.com

Stéphanie Boué^1+^
Email: stephanie.boue@pmi.com

Walter K Schlage^2^
Email: walter.schlage@contracted.pmi.com

Natalia Boukharov^3^
Email: nboukharov@selventa.com

Jurjen W Westra^3^
Email: wwestra@genedata.com

Stephan Gebel^2^
Email: stephan.gebel@uni.lu

Aaron VanHooser^3^
Email: avanhooser@selventa.com

Marja Talikka^1^
Email: marja.talikka@pmi.com

R Brett Fields^3^
Email: bfields@selventa.com

Emilija Veljkovic^1^
Email: emilija.veljkovic@pmi.com

Michael J Peck^1^
Email: michael.peck@pmi.com

Carole Mathis^1^
Email: carole.mathis@pmi.com

Vy Hoang^3^
Email: vhoang@selventa.com

Carine Poussin^1^
Email: carine.poussin@pmi.com

Renee Deehan^3^
Email: rkenney@selventa.com

Katrin Stolle^1^
Email: katrin.stolle@gmx.de

Julia Hoeng^1^
Email: julia.hoeng@pmi.com

Manuel C Peitsch^1^
Email: [manuel.peitsch@pmi.com](mailto:manuel.peitsch@pmi.com)

^1^ Philip Morris International R&D, Philip Morris Products S.A., Quai Jeanrenaud 5, 2000 Neuchâtel, Switzerland

^2^ Philip Morris International R&D, Philip Morris Research Laboratories GmbH, Fuggerstr.3, 51149 Koeln, Germany

^3^ Selventa, One Alewife Center, Cambridge, MA 02140, USA

^+^Equal contributors

**Methods**

**Selventa Knowledgebase**

The nodes and edges comprising the V-IPN were assembled from the Selventa Knowledgebase, a comprehensive repository containing over 1.5 million nodes (biological concepts and entities) and over 7.6 million edges (assertions about causal and non-causal relationships between nodes). The assertions in the Knowledgebase are derived from peer-reviewed scientific literature as well as other public and proprietary databases. Specifically, each assertion describes an individual experimental observation from an experiment performed in a human, mouse, and rat species context, either *in vitro* or *in vivo*. Assertions also capture information about the referring source (e.g., the PubMed ID [PMID] for journal articles listed in MEDLINE), as well as key contextual information including the species and the tissue or cell line from which the experimental observation was derived. An example causal assertion is the increased transcriptional activity of STAT1 (signal transducer and activator of transcription 1) causes an increase in the mRNA expression of ICAM1 (intracellular adhesion molecule 1) [endothelial cell; Human; PMID 21346151] [1]. The Knowledgebase contains causal relationships derived from healthy tissues and disease areas such as inflammation, metabolic disorders, cardiovascular disease, liver injury and cancer.

**Reverse Causal Reasoning (RCR): hypothesis generation process**

RCR analysis of model building datasets was used to generate lists of nodes that were captured as increased or decreased. These lists generated from the building datasets were used to aid in the selection (vetting) of nodes for inclusion in the V-IPN. The lists generated from the test datasets were used for network evaluation (see below). RCR allows identifying potential upstream controllers for the mRNA State Changes in the transcriptomic datasets. These potential upstream controllers are called “HYPs”. Each HYP represents a potential explanation for the subset of State Changes that are causally downstream of the HYP as individual assertions in the Selventa Knowledgebase. HYP generation and the use of causal knowledge in RCR has been described elsewhere [2].

Each HYP was scored according to two probabilistic scoring metrics, richness and concordance. Richness is the probability that the number of observed RNA State Changes connected to a given HYP could have occurred by chance alone, calculated using the hypergeometric distribution. Concordance is the probability that the number of observed mRNA State Changes that match the direction of the HYP (e.g., increased or decreased activity or abundance of a node) could have occurred by chance alone, calculated using a binomial distribution. HYPs meeting both richness and concordance *p*-values <0.05 were considered to be statistically, although not necessarily biologically, significant. For the purposes of network model construction, each scored HYP meeting the minimum statistical cutoffs for richness and concordance was evaluated and selected for integration based on its biological plausibility and relevance to the perturbation and biological context (e.g., cell type).

**Pre-processing of transcriptomics datasets**

Datasets used for building and evaluation are described in Table 1. Briefly, three previously published datasets, GSE2372 and GSE10000 (aortae of apoE-deficient mice at 32 weeks and 78 weeks respectively) [3] and GSE29903 (HCAECs treated with oxidized 1-palmitoyl-2-arachidonoyl-snglycero-3-phosphatidylcholineoxidized [Ox-PAPC]) [4], were used to augment the Literature Model. Three previously published datasets, GSE13139 (GFP-control and LOX-1 overexpressing HCAECs treated with oxLDL) [5], GSE20060 (HCAECs treated with ox-PAPC) [6], GSE40231 (paired clinical samples of atherosclerotic wall of coronary artery and mammary artery from 37 patients) [7], and an internal dataset, E-MTAB-1696 (aortas from ApoE^-/-^ mice exposed for 1 month to mainstream cigarette smoke vs. fresh air control) were used for model evaluation. A dataset from normal human bronchial epithelial (NHBE) cells treated with PD-0332991 (Biozol GmbH, Eching, Germany), a pyrodopyrimidine-derived cyclin-dependent kinase (CDK) inhibitor with high specificity for CDK4 and CDK6, was used as a CV- unrelated negative control (E-MTAB-1272) [8]. Cells were treated with PD-0332991 for 24 hours and cells collected 8 hours after washing with cell culture media.

Public datasets were downloaded from Gene Expression Omnibus (GEO) (<http://www.ncbi.nlm.nih.gov/gds>). Transcriptomics data from NHBE cells is available in ArrayExpress (accession number: E-MTAB-1272). Robust Microarray Analysis (RMA) background correction and quantile normalization were used to generate microarray expression values for GSE2372 (CEL files), while no normalization was used to generate expression values for GSE29903 (log2-transformed data matrix generated from the provided series matrix file), since the array data were originally normalized with the median invariant method using Microarray Suite 5.0 software [4]. An overall linear model was fit to the data for all sample groups, and specific contrasts of interest were evaluated to generate raw *p*-values for each probe set on the expression array [9]. The Benjamini-Hochberg (FDR) method was then used to correct for multiple testing effects. Probe sets were considered to have statistically-significant changed expression levels in a specific comparison if they had an adjusted *p*-value of less than 0.05, an absolute fold change greater than 1.3, and an average expression intensity greater than 150 in either treatment group. NetAffx version na32 feature annotation files, available from Affymetrix (www.Affymetrix.com), were used to map probe sets to genes. In our analysis, genes represented by multiple probe sets were considered to have changed if at least one probe set was observed to change. Gene expression changes that met these criteria are called “State Changes” and have the directional qualities of “increased” or “decreased”, i.e., they were upregulated or downregulated, respectively, in response to the experimental condition. The number of State Changes for each dataset is listed in Table 3.

**V-IPN construction: model structure and boundaries**

Nodes in the V-IPN are biological entities or processes such as mRNA expression and protein abundance, or modified biomolecules (e.g., OxLDL). Network edges are relationships between the nodes categorized as causal or non-causal. Causal edges are directional cause-effect relationships between nodes (e.g., the transcriptional activity of NFκB directly increases the gene expression of VCAM1), whereas non-causal edges connect different forms of a biological entity, such as the protein abundance to its transcriptional activity. Node and edge relationships are described using the Biological Expression Language (BEL), which allows for the semantic representation of relationships and causal inference obtained from prior scientific knowledge. For instance, a change in A causing a change in B, as demonstrated in specific experimental contexts [2]. The model boundaries were defined as biological functions and relationships described for the disease, species (e.g., human), tissues (e.g., aortas) and cells (e.g., ECs) of interest.

The network nodes are modularly integrated and they relate biological processes to experimentally measurable quantities. Driven by analytical algorithms constrained by path length within the network, a set of causal paths, termed HYPs, were generated. This set of causal relationships, connect biological events and effectors (e.g., cell migration) to downstream measurable entities (e.g., gene expression). Differential measurements in a given dataset were then mapped to the entire set of feasible HYPs defined within the network. Each HYP was evaluated as a potential explanation of the observed changes in the experimental dataset. Causal edges linking nodes establish the directionality of changes, which are defined as State Changes and reduced to discrete values (up, down or no change). Causal edges are supported by hundreds of scientific references (PubMed). Through specific cell and disease contexts, a literature model was created via causal connections. RCR of relevant vascular datasets (Table 1) was then used to augment the model (Figure 1A). The resulting integrated version was reviewed by scientists with expertise in vascular biology and inflammation and the final model was evaluated using five additional, independent transcriptomics datasets (Test Datasets, Table 1). To evaluate the relevance of the RCR-generated HYPs, multiple criteria were applied, including the underlying statistical and biological significance in relation to the experimental system of the dataset, the biological evidence linking a given HYP to existing nodes in the literature model, and the consistency of the directionality of the HYPs evaluated by two significance statistics, concordance and richness (see above) [2]. Vital numbers covering the contents of the six subnetworks (nodes, HYPs, edges and statements), is presented in Figure 3. The six subnetworks accompany this manuscript in.xls and XGMML formats (Additional file 3), and can be viewed using freely available network visualization applications such as Cytoscape (http://www.cytoscape.org/).

The V-IPN network was constructed in a sequential process using content from two main sources, nodes and edges derived from prior knowledge described in the scientific literature, and HYP nodes obtained from the computational analysis of transcriptomic profiling data. The causal relationships within the subnetworks were extracted from the Selventa Knowledgebase, a unified collection of over 1.5 million elements of biological knowledge curated from public literature. When causal connections did not exist in the Selventa Knowledgebase, they were identified from public literature and manually curated. The literature model was then augmented with additional HYP nodes derived from the computational analysis of three transcriptomic profiling datasets using RCR (Table 1). Datasets GSE2372 and GSE10000 contained transcriptomic data from the aortas of 32 and 78 week-old ApoE^-/-^ mice, respectively, as compared to their wild-type counterparts (C57BL/6). The third data set (GSE29903) contained information from primary human aortic ECs (HAECs) exposed to oxidized phospholipids, specifically, oxidized 1-palmitoyl-2-arachidonoyl-sn-glycero-3-phosphocholine (oxPAPC), which has been shown to induce endothelial cell dysfunction [10]. Datasets GSE2372 and GSE29903 were used in the construction of all V-IPN subnetworks. GSE10000 was used for the construction of the *Plaque Destabilization* subnetwork. Names describing the species and experimental settings for each dataset were created and they were used throughout the results and discussion section to facilitate comparative analyses.

**ApoE-deficient mice exposed to cigarette smoke**

***Smoke generation and ApoE^-/-^ mice exposure to cigarette smoke.*** The E-MTAB-1696 gene expression dataset was generated from aortas of ApoE^-/-^ mice exposed to cigarette smoke (CS). All animal experimental procedures were conducted at Vivotecnia (Madrid, Spain) in conformity with the American Association for Laboratory Animal Science Policy on the Humane Care and Use of Laboratory Animals [11], and were approved by an Institutional Animal Care and Use Committee (IACUC). Female ApoE^-/-^ (ApoE/Bom, B6.129P2_ApoE ^tm1 Ulnc^ N_11_) mice (Taconic, USA), aged 7 to 10 weeks, were randomly allocated to the experimental groups. Animals were fed a normal chow diet. The University of Kentucky reference research cigarette 3R4F was obtained from the Tobacco Research and Development Center (University of Kentucky, KY, USA). Smoking parameters including puff duration and volume were conducted in conformity with the Health Canada Intense (HCI) smoking regimen [12] as previously described [13]. Total particulate matter (TPM) levels for CS-exposed groups were targeted at 600µg/l. The CS exposure regimen consisted of 4 1-hour periods a day and 30-minute intervals with fresh filtered air, 5 days a week. Sham animals were exposed to fresh-filtered air only using a similar 4 1-hour period regimen.

***Total cholesterol measurements in plasma of ApoE^-/-^ mice.*** Aliquots of plasma from sham and CS-exposed mice were analysed using commercial kits from Thermo Clinical Labsystems (Frankfurt, Germany) according to the manufacturer’s instructions.

***Atherosclerotic plaque measurements in the aortic arch of ApoE^-/-^ mice.*** Mice were anesthetized (pentobarbital sodium, 100 mg/kg body weight, i.p.), exsanguinated, and perfused with ice-cold phosphate buffered saline (PBS). After careful removal of the aortic arch with the aid of a dissecting microscope, tissue was opened longitudinally and fixed with dissecting pins. Pictures of the opened aortic arch were taken with a TBK Digital camera KY-F70 (Leica) and images were analysed with DISKUS® image analysis software (Hilgers, Koenigswinter, Germany). The intimal area covered by plaques was determined and normalized to the whole aortic arch area.

***Immunohistochemical stainings.*** A segment of aorta close to the aortic root from sham and CS-exposed mice was dissected, fixed and embedded in OCT. Frozen sections were obtained and stained with H&E and an antibody raised against MAC-3, an antigen expressed on mouse mononuclear phagocytes.

***Aortic arch gene expression profiling.*** RNA was isolated from cryopreserved aortic arch following the manufacturer’s recommendations in the GeneChip(R) HT 3' IVT Express Kit (Santa Clara, CA) guide. Gene expression profiles were acquired on Affymetrix GeneChip Mouse Genome 430 2.0 arrays according to manufacturer’s recommendations. Raw RNA expression data for each data set were analysed using the affy and limma [9,14] packages of the Bioconductor suite of microarray analysis tools [14] available for the R statistical environment [15]. Robust Microarray Analysis (RMA) background correction and quantile normalization were used to generate microarray expression values [16]. An overall linear model was fit to the data for all sample groups, and specific contrasts of interest were evaluated to generate raw *p*-values for each probe set on the expression array [99]. The Benjamini-Hochberg False Discovery Rate (FDR) method was then used to correct for multiple testing effects. Microarray data were submitted to ArrayExpress (E-MTAB-1696)*).*

**References**

1. Sikorski K, Chmielewski S, Przybyl L, Heemann U, Wesoly J, Baumann M, Bluyssen HA: **STAT1-mediated signal integration between IFNgamma and LPS leads to increased EC and SMC activation and monocyte adhesion.** *American journal of physiology Cell physiology* 2011, **300:**C1337-1344.

2. Catlett NL, Bargnesi AJ, Ungerer S, Seagaran T, Ladd W, Elliston KO, Pratt D: **Reverse causal reasoning: applying qualitative causal knowledge to the interpretation of high-throughput data.** *BMC Bioinformatics* 2013, **14:**340.

3. Grabner R, Lotzer K, Dopping S, Hildner M, Radke D, Beer M, Spanbroek R, Lippert B, Reardon CA, Getz GS, Fu YX, Hehlgans T, Mebius RE, van der Wall M, Kruspe D, Englert C, Lovas A, Hu D, Randolph GJ, Weih F, Habenicht AJ: **Lymphotoxin beta receptor signaling promotes tertiary lymphoid organogenesis in the aorta adventitia of aged ApoE-/- mice.** *The Journal of experimental medicine* 2009, **206:**233-248.

4. Gargalovic PS, Imura M, Zhang B, Gharavi NM, Clark MJ, Pagnon J, Yang WP, He A, Truong A, Patel S, Nelson SF, Horvath S, Berliner JA, Kirchgessner TG, Lusis AJ: **Identification of inflammatory gene modules based on variations of human endothelial cell responses to oxidized lipids.** *Proceedings of the National Academy of Sciences of the United States of America* 2006, **103:**12741-12746.

5. Mattaliano MD, Huard C, Cao W, Hill AA, Zhong W, Martinez RV, Harnish DC, Paulsen JE, Shih HH: **LOX-1-dependent transcriptional regulation in response to oxidized LDL treatment of human aortic endothelial cells.** *American journal of physiology Cell physiology* 2009, **296:**C1329-1337.

6. Romanoski CE, Lee S, Kim MJ, Ingram-Drake L, Plaisier CL, Yordanova R, Tilford C, Guan B, He A, Gargalovic PS, Kirchgessner TG, Berliner JA, Lusis AJ: **Systems genetics analysis of gene-by-environment interactions in human cells.** *American journal of human genetics* 2010, **86:**399-410.

7. Hagg S, Skogsberg J, Lundstrom J, Noori P, Nilsson R, Zhong H, Maleki S, Shang MM, Brinne B, Bradshaw M, Bajic VB, Samnegard A, Silveira A, Kaplan LM, Gigante B, Leander K, de Faire U, Rosfors S, Lockowandt U, Liska J, Konrad P, Takolander R, Franco-Cereceda A, Schadt EE, Ivert T, Hamsten A, Tegner J, Bjorkegren J: **Multi-organ expression profiling uncovers a gene module in coronary artery disease involving transendothelial migration of leukocytes and LIM domain binding 2: the Stockholm Atherosclerosis Gene Expression (STAGE) study.** *PLoS Genet* 2009, **5:**e1000754.

8. Belcastro V, Poussin C, Gebel S, Mathis C, Schlage WK, Lichtner RB, Quadt-Humme S, Wagner S, Hoeng J, Peitsch MC: **Systematic verification of upstream regulators of a computable cellular proliferation network model on non-diseased lung cells using a dedicated dataset.** *Bioinformatics and biology insights* 2013, **7:**217-230.

9. Smyth GK: **Linear models and empirical bayes methods for assessing differential expression in microarray experiments.** *Stat Appl Genet Mol Biol* 2004, **3:**Article3.

10. Starosta V, Wu T, Zimman A, Pham D, Tian X, Oskolkova O, Bochkov V, Berliner JA, Birukova AA, Birukov KG: **Differential regulation of endothelial cell permeability by high and low doses of oxidized 1-palmitoyl-2-arachidonyl-sn-glycero-3-phosphocholine.** *Am J Respir Cell Mol Biol* 2012, **46:**331-341.

11. Potkay S, Garnett NL, Miller JG, Pond CL, Doyle DJ: **Frequently asked questions about the Public Health Service Policy on Humane Care and Use of Laboratory Animals.** *Contemporary topics in laboratory animal science / American Association for Laboratory Animal Science* 1997, **36:**47-50.

12. Burns DM, Dybing E, Gray N, Hecht S, Anderson C, Sanner T, O'Connor R, Djordjevic M, Dresler C, Hainaut P, Jarvis M, Opperhuizen A, Straif K: **Mandated lowering of toxicants in cigarette smoke: a description of the World Health Organization TobReg proposal.** *Tobacco control* 2008, **17:**132-141.

13. Boue S, Tarasov K, Janis M, Lebrun S, Hurme R, Schlage W, Lietz M, Vuillaume G, Ekroos K, Steffen Y, Peitsch MC, Laaksonen R, Hoeng J: **Modulation of atherogenic lipidome by cigarette smoke in apolipoprotein E-deficient mice.** *Atherosclerosis* 2012, **225:**328-334.

14. Gentleman RC, V.J.C., Bates DM, Bolstad B, Dettling M, Dudoit S, Ellis B, Gautier L, Ge Y, Gentry J, Hornik K, Hothorn T, Huber W, Iacus S, Irizarry R, Leisch F, Li C, Maechler M, Rossini AJ, Sawitzki G, Smith C, Smyth G, Tierney L, Yang JYH, Zhang J: **Bioconductor: Open software development for computational biology and bioinformatics.** *Genome Biology* 2004, **5:**R80.

15. R Development Core Team: **R: A Language and Environment for Statistical Computing.** 2007.

16. Irizarry RA, B.H., Collin F, Beazer-Barclay YD, Antonellis KJ, Scherf U, Speed TP: **Exploration, normalization, and summaries of high density oligonucleotide array probe level data.** *Biostatistics* 2003, **4:**249-264.
