# Supplementary figures and images for: A vascular biology network model focused on inflammatory processes to investigate atherogenesis and plaque instability
Source: J Transl Med. 2014 Jun 26;12:185. doi: 10.1186/1479-5876-12-185 (PMC4227037; doi:10.1186/1479-5876-12-185)

## Slide 1
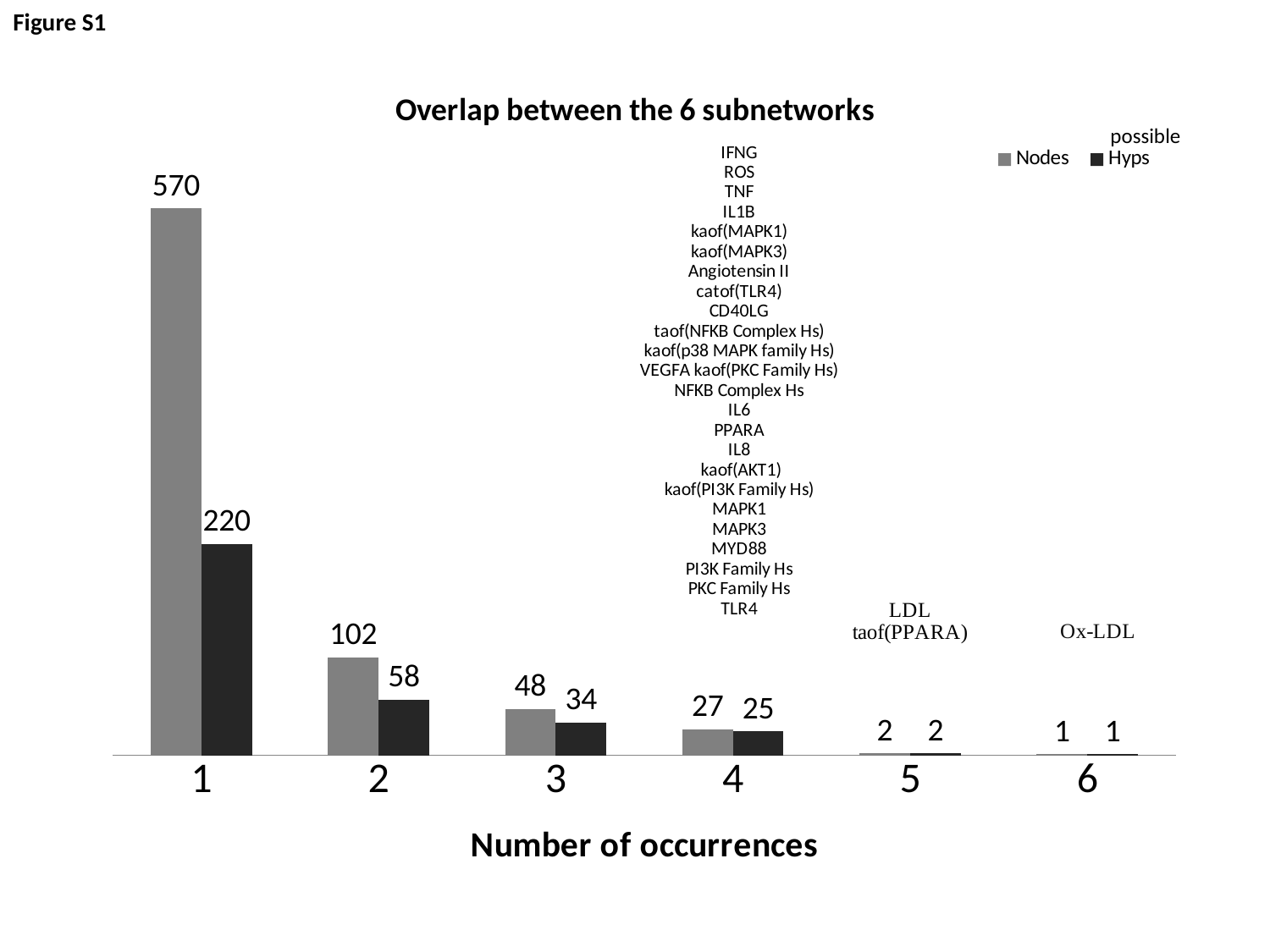

Figure S1
### Chart: Overlap between the 6 subnetworks
| Category | | |
|---|---|---|possible

Supplement: Additional file 4: Figure S1 — Frequency rate of nodes and HYPs across the six subnetworks. As the number of events increases, the frequency of those occurrences in all networks decreases. Twenty five HYPs were present in all subnetworks at a simultaneous event rate of 4, whereas a single HYP, Ox-LDL, was present once in all networks. [file 1479-5876-12-185-S4.pptx]

A

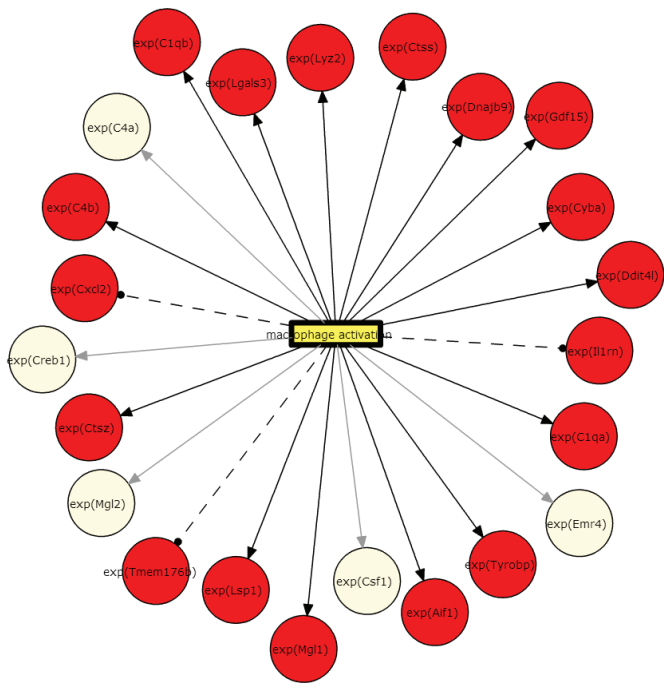

B

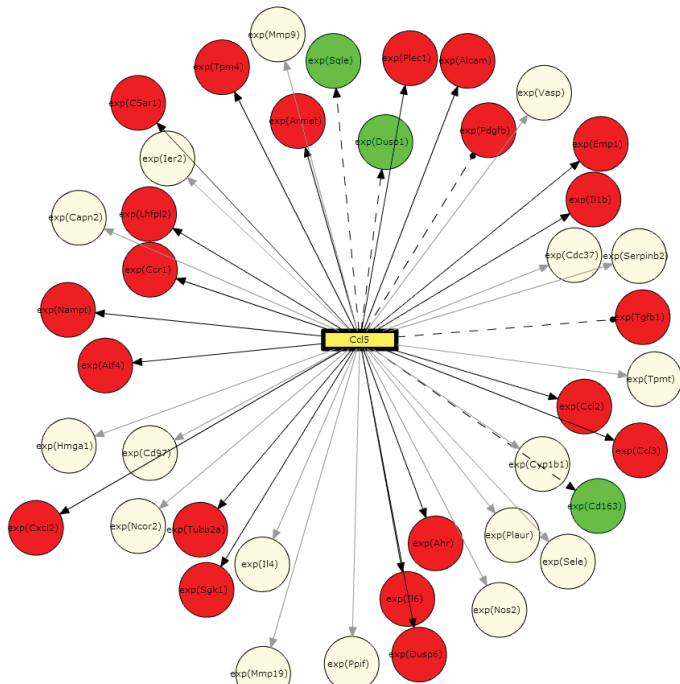

C

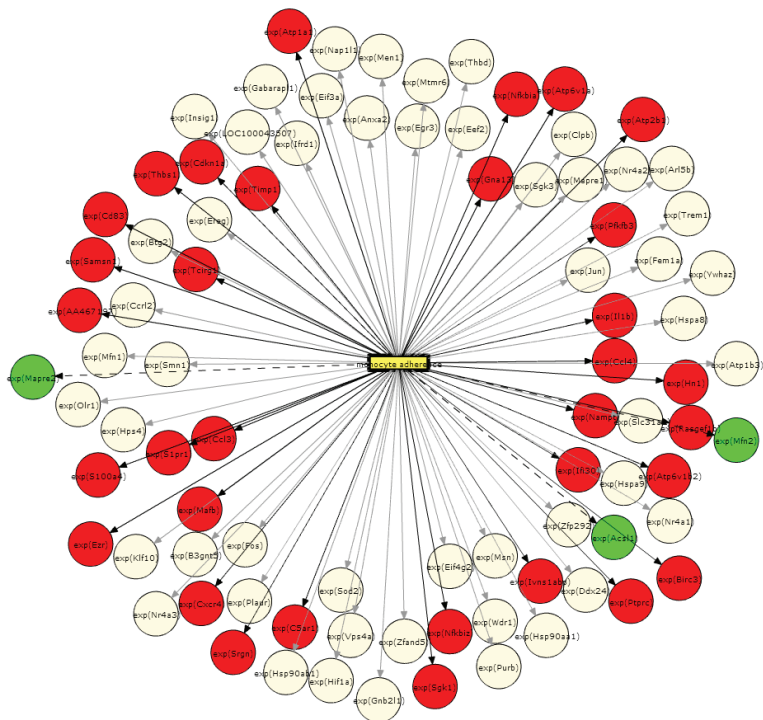

D

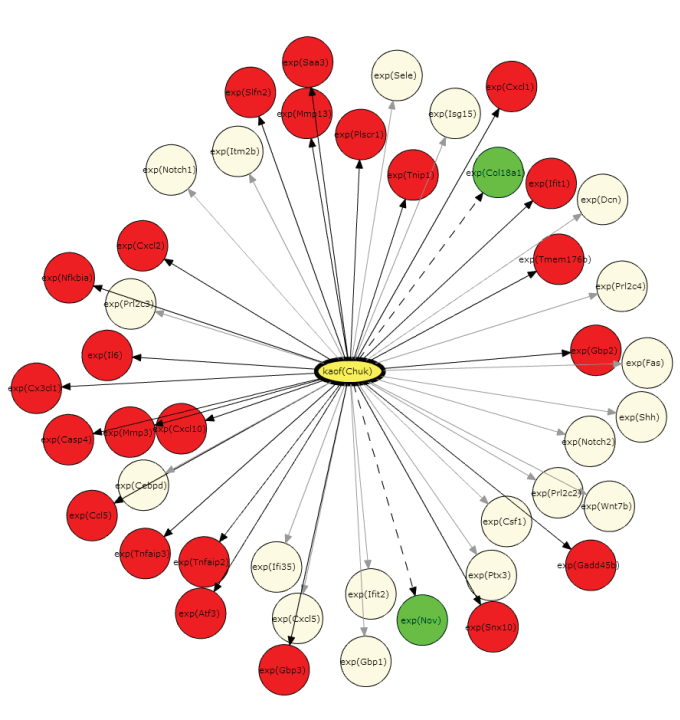

Supplement: Additional file 8: Figure S3 — A. The HYP with the upstream node macrophage activation scored for the Mm_Ao_78w_ApoE_vs_wt dataset. This HYP contains 23 measured RNA abundance nodes, represented as circles colored by differential expression (red = significantly increased, green = significantly decreased, white = no significant change). A total of 18 differentially expressed RNAs mapped to the HYP network, including 15 supporting increased mechanism activity (solid arrows) and three supporting decreased activity (dotted lines). B. The HYP with the upstream node Ccl5 scored for the Mm_Ao_78w_ApoE_vs_wt dataset. This HYP contains 41 measured RNA abundance nodes, represented as circles colored by differential expression (red = significantly increased, green = significantly decreased, white = no significant change). A total of 24 differentially expressed RNAs mapped to the HYP network, including 19 supporting increased mechanism activity (solid arrows) and five supporting decreased activity (dotted lines). C. The HYP with the upstream node monocyte adherence, scored for the Mm_Ao_78w_ApoE_vs_wt dataset. This HYP contains 87 measured RNA abundance nodes, represented as circles colored by differential expression (red = significantly increased, green = significantly decreased, white = no significant change). A total of 36 differentially expressed RNAs mapped to the HYP network, including 33 supporting increased mechanism activity (solid arrows) and three supporting decreased activity (dotted lines). D. The HYP with the upstream node kaof(Chuk), scored for the Mm_Ao_78w_ApoE_vs_wt dataset. This HYP contains 44 measured RNA abundance nodes, represented as circles colored by differential expression (red = significantly increased, green = significantly decreased, white = no significant change). A total of 25 differentially expressed RNAs mapped to the HYP network, including 23 supporting increased mechanism activity (solid arrows) and two supporting decreased activity (dotted lines). [file 1479-5876-12-185-S8.pdf]

## Slide 1
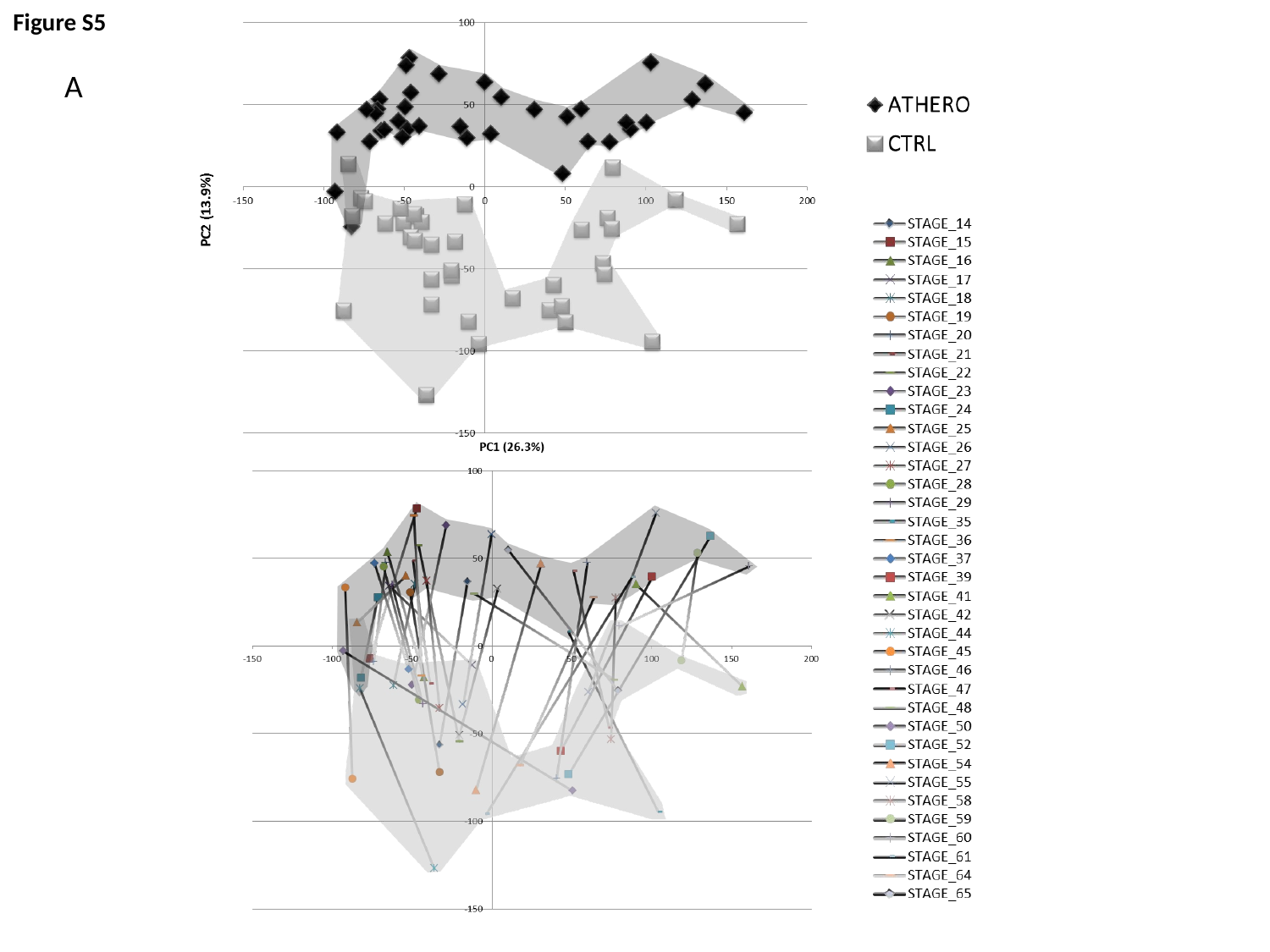

Figure S5
A

Supplement: Additional file 13: Figure S5 — Principal component analysis (PCA) of samples from Hs_athCA_vs_ctIMA (GSE40231). PCA plot A illustrates the principal components of the gene expression profiles of 37 pairs of samples from the atherosclerotic coronary arteries and control internal mammary arteries from the STAGE study. Although the separation between atherosclerotic tissue and control mammary artery is relatively clear, the pairing of the samples (each pair from one patient) empowers the downstream analysis as illustrated when looking at the distance between the pairs of samples. PCA plot B highlights the relationships between the paired samples, demonstrating that for the samples that may look at the borderline between the two groups (ATHERO and CTRL), the difference between the atherosclerotic vessel and its control artery is still very clear, and in the same direction as for the other pairs. [file 1479-5876-12-185-S13.pptx]
