# Supplementary material for: A vascular biology network model focused on inflammatory processes to investigate atherogenesis and plaque instability
Source: J Transl Med. 2014 Jun 26;12:185. doi: 10.1186/1479-5876-12-185 (PMC4227037; doi:10.1186/1479-5876-12-185)
Supplement: Additional file 5: Figure S2 — Node overlap between subnetworks. Table A shows the number of overlapping nodes between all six of the individual subnetworks. Table B shows, as a percentage, the degree of node overlap between the six subnetworks. Colored cells reflect the degree of overlap from low (dark blue) to high degrees of overlap (dark red). [file 1479-5876-12-185-S5.pptx]

## Slide 1
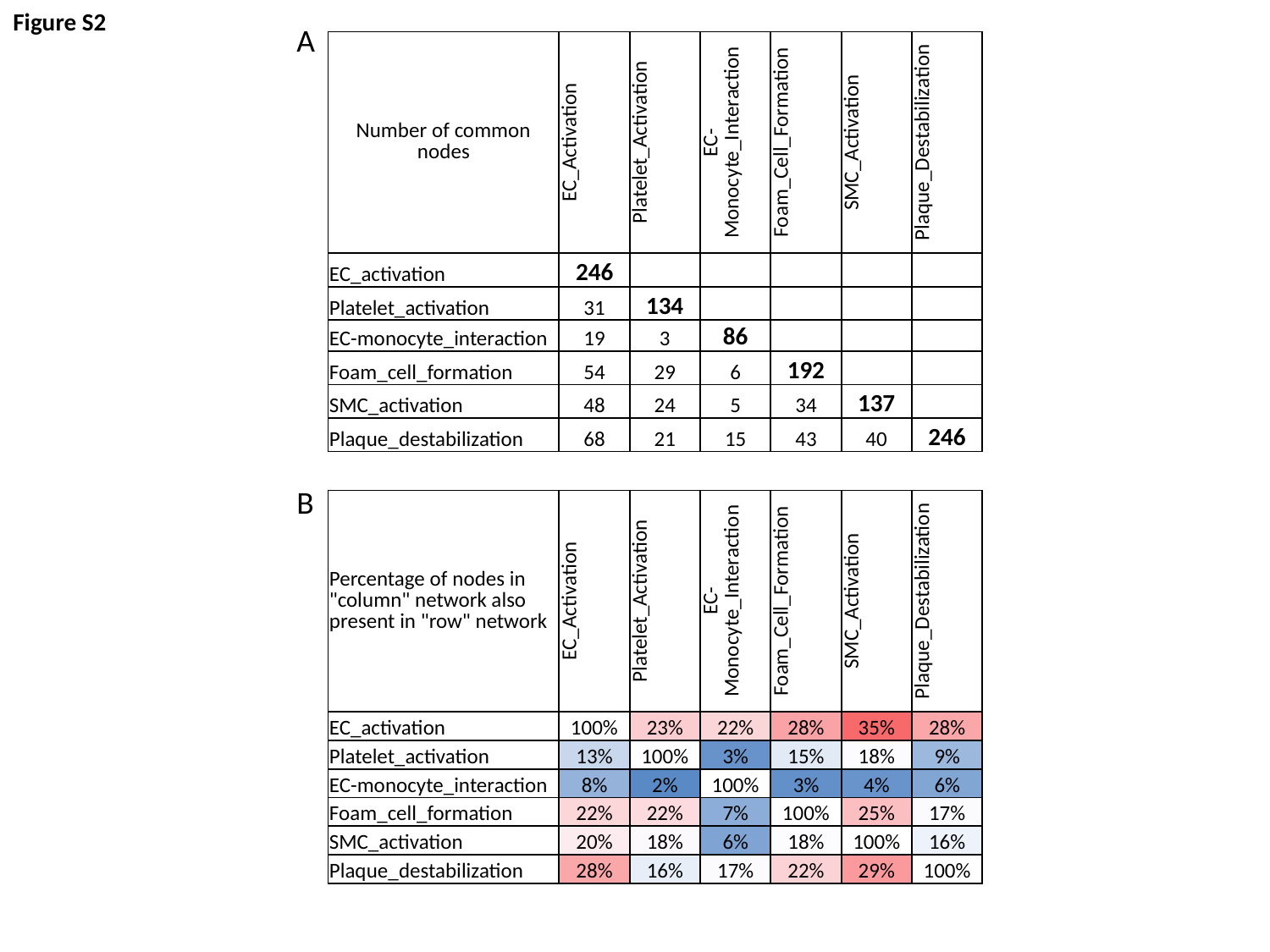

Figure S2
A
| Number of common nodes | EC\_Activation | Platelet\_Activation | EC-Monocyte\_Interaction | Foam\_Cell\_Formation | SMC\_Activation | Plaque\_Destabilization |
| --- | --- | --- | --- | --- | --- | --- |
| EC\_activation | 246 | | | | | |
| Platelet\_activation | 31 | 134 | | | | |
| EC-monocyte\_interaction | 19 | 3 | 86 | | | |
| Foam\_cell\_formation | 54 | 29 | 6 | 192 | | |
| SMC\_activation | 48 | 24 | 5 | 34 | 137 | |
| Plaque\_destabilization | 68 | 21 | 15 | 43 | 40 | 246 |
B
| Percentage of nodes in "column" network also present in "row" network | EC\_Activation | Platelet\_Activation | EC-Monocyte\_Interaction | Foam\_Cell\_Formation | SMC\_Activation | Plaque\_Destabilization |
| --- | --- | --- | --- | --- | --- | --- |
| EC\_activation | 100% | 23% | 22% | 28% | 35% | 28% |
| Platelet\_activation | 13% | 100% | 3% | 15% | 18% | 9% |
| EC-monocyte\_interaction | 8% | 2% | 100% | 3% | 4% | 6% |
| Foam\_cell\_formation | 22% | 22% | 7% | 100% | 25% | 17% |
| SMC\_activation | 20% | 18% | 6% | 18% | 100% | 16% |
| Plaque\_destabilization | 28% | 16% | 17% | 22% | 29% | 100% |
