# Supplementary material for: A vascular biology network model focused on inflammatory processes to investigate atherogenesis and plaque instability
Source: J Transl Med. 2014 Jun 26;12:185. doi: 10.1186/1479-5876-12-185 (PMC4227037; doi:10.1186/1479-5876-12-185)
Supplement: Additional file 6: Table S1 — Common HYP coverage of V-IPN and cell proliferation subnetworks by a dataset from NHBE cells (Hs_NHBE_CDKinh_rel_vs_blk_8h) used as a negative control. (↑) predicted increased, (↓) predicted decreased. [file 1479-5876-12-185-S6.doc]

| **Table S1.** Common HYP coverage of V-IPN and cell proliferation subnetworks by a dataset from NHBE cells *(Hs_NHBE_CDKinh_rel_vs_blk_8h)* used as a negative control. (🡩) predicted increased, (🡫) predicted decreased | | | | |
| --- | --- | --- | --- | --- |
| HYPs | V-IPN | | Cell Proliferation Network | |
|  | EC Activation | SMC Activation | Cell Cycle | Growth Factor |
| gtpof(RHOA) (🡫) | 1 | 1 | 1 | 0 |
| taof(NFKB Complex Hs) (🡩) | 1 | 1 | 0 | 1 |
| kaof(PI3K Family Hs) (🡫) | 1 | 1 | 0 | 1 |
| kaof(MAPK3) (🡩) | 1 | 1 | 0 | 1 |
| taof(STAT3) (🡩) | 1 | 0 | 1 | 1 |
| CDKN1A (🡩) | 1 | 0 | 1 | 0 |
| VEGFA (🡩) | 1 | 0 | 0 | 1 |
| paof(PTEN) (🡩) | 1 | 0 | 0 | 1 |
| taof(EP300) (🡫) | 1 | 0 | 0 | 1 |
| MYC (🡫) | 0 | 1 | 1 | 1 |
| taof(RB1) (🡩) | 0 | 1 | 1 | 1 |
| CCND1 (🡫) | 0 | 1 | 1 | 0 |
| E2F1 (🡫) | 0 | 1 | 1 | 0 |
| taof(E2F1) (🡫) | 0 | 1 | 1 | 0 |
| CDK4 (🡫) | 0 | 1 | 1 | 0 |
| taof(HIF1A) (🡩) | 0 | 1 | 0 | 1 |
