# Supplementary material for: A vascular biology network model focused on inflammatory processes to investigate atherogenesis and plaque instability
Source: J Transl Med. 2014 Jun 26;12:185. doi: 10.1186/1479-5876-12-185 (PMC4227037; doi:10.1186/1479-5876-12-185)
Supplement: Additional file 9: Figure S4 — Coverage and OR of the dataset Hs_athCA_vs_ctIMA (GSE40231) across other network models. Subnetworks with less than 10 HYPs were not included. IPN: Inflammatory Process Network, TRAG: Tissue Repair and Angiogenesis. DACS: DNA damage, Autophagy, Cell death (apoptosis and necroptosis), and Senescence. [file 1479-5876-12-185-S9.pptx]

## Slide 1
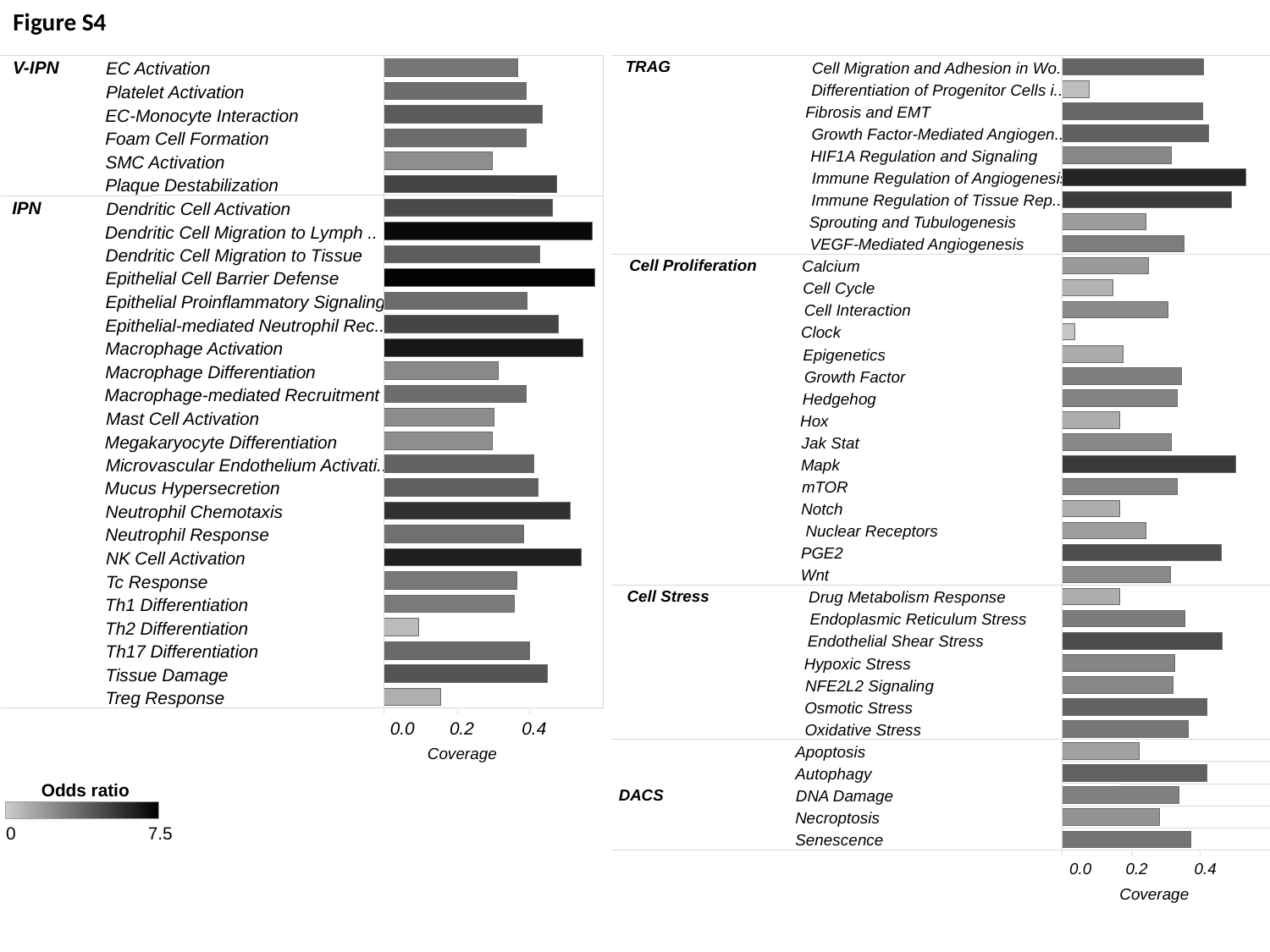

Figure S4
TRAG
Cell Migration and Adhesion in Wo..
Differentiation of Progenitor Cells i..
Fibrosis and EMT
Growth Factor-Mediated Angiogen..
HIF1A Regulation and Signaling
Immune Regulation of Angiogenesis
Immune Regulation of Tissue Rep..
Sprouting and Tubulogenesis
VEGF-Mediated Angiogenesis
Cell Proliferation
Calcium
Cell Cycle
Cell Interaction
Clock
Epigenetics
Growth Factor
Hedgehog
Hox
Jak Stat
Mapk
mTOR
Notch
Nuclear Receptors
PGE2
Wnt
Cell Stress
Drug Metabolism Response
Endoplasmic Reticulum Stress
Endothelial Shear Stress
Hypoxic Stress
NFE2L2 Signaling
Osmotic Stress
Oxidative Stress
Apoptosis
Autophagy
DACS
DNA Damage
Necroptosis
Senescence
0.0
0.2
0.4
Coverage
V-IPN
EC Activation
Platelet Activation
EC-Monocyte Interaction
Foam Cell Formation
SMC Activation
Plaque Destabilization
IPN
Dendritic Cell Activation
Dendritic Cell Migration to Lymph ..
Dendritic Cell Migration to Tissue
Epithelial Cell Barrier Defense
Epithelial Proinflammatory Signaling
Epithelial-mediated Neutrophil Rec..
Macrophage Activation
Macrophage Differentiation
Macrophage-mediated Recruitment
Mast Cell Activation
Megakaryocyte Differentiation
Microvascular Endothelium Activati..
Mucus Hypersecretion
Neutrophil Chemotaxis
Neutrophil Response
NK Cell Activation
Tc Response
Th1 Differentiation
Th2 Differentiation
Th17 Differentiation
Tissue Damage
Treg Response
0.0
0.2
0.4
Odds ratio
0
7.5
Coverage
