# Supplementary material for: A vascular biology network model focused on inflammatory processes to investigate atherogenesis and plaque instability
Source: J Transl Med. 2014 Jun 26;12:185. doi: 10.1186/1479-5876-12-185 (PMC4227037; doi:10.1186/1479-5876-12-185)
Supplement: Additional file 10: Table S2 — Transcriptomics-based evaluation of the effects of oxidative stimuli on primary HAEC cultures vs. immortalized HAECs. [file 1479-5876-12-185-S10.doc]

| **Table S2.** Transcriptomics-based evaluation of the effects of oxidative stimuli on primary HAEC cultures vs. immortalized HAECs. | | | | | | | | | |
| --- | --- | --- | --- | --- | --- | --- | --- | --- | --- |
| *Hs_EC_GFP_oxLDL_vs_ct* | *Hs_EC_LOX1_oxLDL_vs_ct* | *Hs_EC_oxPAP_vs_ct* | N^o^ of HYPs | *EC_Activation* | *Platelet_Activation* | *EC-Monocyte_Interaction* | *Foam_Cell_Formation* | *SMC_Activation* | *Plaque_Destabilization* |
| X | X | X | 12 | 6 (4) | 1 (2) | 2 (7) | 5 (4) | 2 (2) | 3 (3) |
| X | X | - | 8 | 5 (3) | 3 (5) | 2 (7) | 5 (4) | 3 (3) | 5 (5) |
| X | - | X | 6 | 3 (2) | 0 (0) | 0 (0) | 3 (3) | 4 (5) | 3 (3) |
| - | X | X | 18 | 12 (8) | 3 (5) | 3 (10) | 3 (3) | 5 (6) | 8 (8) |
| X | - | - | 5 | 2 (1) | 0 (0) | 0 (0) | 3 (3) | 0 (0) | 3 (3) |
| - | X | - | 14 | 7 (5) | 1 (2) | 1 (3) | 8 (7) | 6 (7) | 5 (5) |
| - | - | X | 41 | 24 (15) | 8 (14) | 3 (10) | 10 (8) | 12 (14) | 13 (13) |
| - | - | - | 639 | 96 (62) | 43 (73) | 19 (63) | 81 (69) | 56 (64) | 64 (62) |
| Numbers between parentheses are percentage of total number of possible HYPs. | | | | | | | | | |
