# Supplementary material for: A vascular biology network model focused on inflammatory processes to investigate atherogenesis and plaque instability
Source: J Transl Med. 2014 Jun 26;12:185. doi: 10.1186/1479-5876-12-185 (PMC4227037; doi:10.1186/1479-5876-12-185)
Supplement: Additional file 11: Table S3 — DAVID functional clustering of common HYPs between Mm_Ao_78w_ApoE_vs_wt and Hs_athCA_vs_ctIMA. (↑) predicted increased in both datasets; (↓) predicted decreased in both datasets. [file 1479-5876-12-185-S11.doc]

| **Table S3.** DAVID functional clustering of common HYPs between *Mm_Ao_78w_ApoE_vs_wt* and *Hs_athCA_vs_ctIMA.* (🡩) predicted increased in both datasets; (🡫) predicted decreased in both datasets. | |
| --- | --- |
| Function | HYPs |
| Inflammatory Response | CCL5 (🡩), catof(F2) (🡩), IL1B (🡩), IL17A (🡩), IL6 (🡩), catof(TLR2) (🡩), TNFRSF1A (🡩) |
| Cytokine activity | CCL5 (🡩), CSF1 (🡩), CSF2 (🡩), IFNB1 (🡩), IFNG (🡩), IL1B (🡩),  IL17A (🡩), IL6 (🡩) |
| Response to organic substance | CCL5 (🡩), IFNG (🡩), taof(IRF3) (🡩), catof(SOD1) (🡫), taof(STAT1) (🡩), catof(TLR2) (🡩) |
| Toll-like receptor signaling pathway | IFNB1 (🡩), taof(IRF3) (🡩), IL1B (🡩), IL6 (🡩), taof(STAT1) (🡩),  catof(TLR2) (🡩) |
| Regulation of transcription | IFNG (🡩), taof(IRF3) (🡩), IL17A (🡩), IL6 (🡩), taof(NCOR1) (🡫), taof(STAT1) (🡩), catof(TLR2) (🡩), TNFRSF1A (🡩), VEGFA (🡩) |
| Cell proliferation | kaof(SRC) (🡩), IL6 (🡩), VEGFA (🡩) |
| Chemotaxis | CCL5 (🡩), IFNG (🡩) |
| X: protein abundance of gene X, catof(X): catalytic activity of protein X, kaof(X): kinase activity of protein X, taof : transcriptional activity of protein X. | |
