# Supplementary material for: A vascular biology network model focused on inflammatory processes to investigate atherogenesis and plaque instability
Source: J Transl Med. 2014 Jun 26;12:185. doi: 10.1186/1479-5876-12-185 (PMC4227037; doi:10.1186/1479-5876-12-185)
Supplement: Supplementary file 23 [file 1479-5876-12-185-S12.doc]

Table S4. Summary of findings and insights provided by each dataset evaluated by RCR and the V-IPN.

	Dataset (ID)	V-IPN Coverage / OR	Experimental Perturbation	Primary Findings	Pathobiological Relevance / Insights	HYPs uniquely predicted in murine and human datasets
Model Building Dataset	Mm_Ao_78w_ApoE_vs_wt (GSE10000)	0.33 / 2.44	Advanced atherosclerotic lesions in aortas of old ApoE-/- mice (78 weeks)	High mechanistic overlapp with human coronary arteries except on the Platelet Activation subnetwork	1) Striking mechanistic similarities in most subnetworks (except Platelet Activation) with human dataset confirms that the ApoE-/- mouse is a robust model of human atherogenesis<br>2) Lack of overlapping between advanced murine and human lesions on platelet function suggests that the ApoE-/- mouse is not a relevant model to investigate plaque instability and rupture of advanced human atherosclerotic lesions	ADAM17, Aldosterone, ATF4, catof(CCR3), catof(CD40), catof(EDNRA), catof(EDNRB), Ceramide, Cholesterol, CSF3, CXCL2, E2F1, EDN1, Estradiol-17beta, gtpof(RHOA), HEXIM1, Histamine, ICAM1, IL1A, JAK2, kaof(EIF2AK3), kaof(IkappaB kinase complex Hs), kaof(IKBKB), kaof(MAP3K1), LDL, LTBR, MYD88, paof(PTEN), PDGF Family Hs, PTK2, RAC1, REL, RELA, SOCS2, STAT1, taof(CEBPD), taof(CREB1), taof(E2F1), taof(ELK1), taof(EP300), taof(IRF5), taof(NR3C2), taof(RB1), TRAF2
Test Datasets	Mm_Ao_16w_ApoE_CS_vs_sham (E-MTAB-1696)	0.16 / 1.75	Early atherosclerotic lesions in aortas of young mice (16 wekks) after 30-day exposure to CS	Low mechanistic overlap with advanced vascular lesions of older ApoE-/- mice	1) ApoE-/- model offers the experimental advantage to studying early atherogenic mechanisms<br>2) ApoE-/- mouse model suitable to studying CS-mediated vascular pathobiology	catof(PTGER3), CAV1, CD40LG, EIF2AK3, gtpof(RAC1), kaof(ERK family Hs), PPARA, PGE2, SCAP, SIRT1, SREBF1, SYK, taof(CTNNB1), taof(EGR1), taof(GATA6), taof(RELA), taof(SREBF1C), taof(SREBF2)
	Hs_EC_GFP_oxLDL_vs_ct (GSE13139)	0.09 / 1.62	Ox-LDL treatment of an immortalized EC line (HAECs from one donor; cells immortalized by SV40)	Low mechanistic overlap (all subnetworks) with human primary ECs	Immortalized cell lines are not a reliable model to investigate genome-wide gene expression changes	---
	Hs_EC_LOX1_oxLDL_vs_ct (GSE13139)	0.15 / 1.99	See above	See above	See above	---
	Hs_EC_oxPAP_vs_ct (GSE20060)	0.23 / 2.32	Ox-PAPC treatment of human aortic ECs obtained from 96 donors	Highest coverage and OR exhibited by any dataset obtained from ECs	In vitro experiments conducted with primary ECs from multiple donors better recreates the biology of cells in intact tissues	---
	Hs_athCA_vs_ctIMA (GSE40231)	0.24 / 2.47	Coronary arteries from CAD patients collected at time of bypass surgery	Highest coverage and OR exhibited by any dataset (except *Mm_Ao_78w_ApoE_vs_wt* , a model construction dataset)	1) The large set of causal mechanisms shared with a murine dataset from advanced lesions (78 week-old ApoE-/- mice) indicates that similar mechanisms underlie the development of advanced atherosclerotic lesions in humans and mice<br>2) The largest coverage and OR scored by this dataset in the Platelet Activation subnetwork highlights the essential role that platelets and thrombus formation play in advanced human lesions	ABCG1, ANGPT1, Arachidonate, catof(F2R), CDKN2A Cortisol, EGR1, GATA6, IFNA1, kaof(AKT Family Hs), kaof(IRAK4), kaof(MAP2K6), kaof(MAPK1), kaof(MAPK3), kaof(MAPK8), kaof(MEK Family Hs), kaof(PI3K Family Hs), LDLR, MAP2K4, Ox-HDL, Ox-LDL, PPARG, taof(CEBPA)
Negative Controls	Hs_NHBE_CDKinh_rel_vs_blk_8h (E-MTAB-1272)	0.16 / 1.39	Exposure to CDK4/6 inhibitor	Low coverage and OR. Low mechanistic overlapping with all other datasets	The V-IPN is highly specific for inflammatory processess underlying vascular biology	---
	Hs_MVEC-C_hpx_vs_ct_24h (GSE11341)	0.14 / 2.20	24h hypoxia (1% O2)	See above	See above	---
	Hs_MVEC-L_hpx_vs_ct_24h (GSE11341)	0.12 / 1.73	24h hypoxia (1% O2)	See above	See above	---
	Hs_JurkT_ars_vs_ct (GSE46909)	0.09 / 1.55	Arsenic trioxide exposure	See above	See above	---

CAD: coronary artery disease; ECs: endothelial cells; HAECs: human aortic endothelial cells (ECs); HCAECs: human coronary artery ECs; GFP: green fluorescent protein; OxPAPC: oxidized 1-palmitoyl-2-arachidonoyl-sn-glycero-3-phosphocholine; oxLDL: oxidized low density lipoprotein; Hs: homo sapiens; IMA: internal mammary artery; Mm: mus musculus, MVECs: microvascular endothelial cells; NHBE: normal human bronchial epithelial cells; h: hours; d: days; wk: weeks; yr: years.
